# A Computational System-Level Evaluation of a Multi-Octave Omnidirectional Direction-Finding System Using Full-Wave Electromagnetic Modeling of a Biconical Array and High-Fidelity Digital Receiver Simulation

**DOI:** 10.3390/s26216852

**Published:** 2026-10-29

**Authors:** Chen Wu, Nandha Dileep, Matthew Ruddick

**Affiliations:** Defence Research and Development Canada–Ottawa Research Centre, Ottawa, ON K1A 0Z4, Canada; nandha.dileep@drdc-rddc.gc.ca (N.D.); matthew.ruddick@drdc-rddc.gc.ca (M.R.)

**Keywords:** full-wave electromagnetic numerical modeling, direction-finding, direction of arrival, angle of arrival, electronic support measures, radar electronic warfare, ultra-wideband array, omnidirectional array, deep neural network, digital receiver

## Abstract

**Highlights:**

A system-level evaluation of a multi-octave, two-dimensional omnidirectional direction-finding (DF) system was conducted using high-fidelity modeling and simulation. The system incorporates a deep neural network-based multi-octave omnidirectional DF framework (DNN-based MOOD-DF) and was evaluated using high-fidelity datasets generated through full-wave electromagnetic modeling of a seven-element three-dimensional biconical antenna array and high-fidelity digital receiver simulation. Three representative frequency bands (2–4 GHz, 10–12 GHz, and 16–18 GHz) were investigated to establish a bridge between previous idealized studies and future hardware prototyping and experimental validation.

**What are the main findings?**

- The integrated modeling and evaluation framework demonstrates that the proposed DF system, incorporating the DNN-based MOOD-DF framework, maintains robust omnidirectional 2D DF performance in the presence of realistic antenna array effects, including mutual coupling, embedded-element patterns, impedance mismatches, and receiver-channel impairments.
- Across the investigated frequency bands, the proposed DF system achieves sub-degree azimuth RMSE even at 0 dB SNR. Elevation RMSE is near sub-degree at a 10 dB SNR and remains below approximately 1.8° at SNRs near 0 dB. The DF system also maintains microsecond-scale inference latency suitable for real-time applications. Potential approaches for further improving elevation-angle estimation accuracy are discussed.
- Benchmark comparisons with CRLB and MUSIC provide theoretical and conventional references for assessing system performance. The results indicate that the proposed DF system can successfully operate on the complex and highly spatially aliased three-dimensional manifold of the 3D multi-octave biconical array, whereas conventional narrowband DF techniques such as MUSIC encounter substantial challenges in this environment.
- The proposed DF system does not require calibration tables or explicit channel-matching procedures during estimation, thereby simplifying deployment compared with conventional direction-finding implementations.

**What are the implications of the main findings?**

- The results represent an important step toward realizing practical multi-octave omnidirectional 2D DF systems by extending validation from idealized array models to a high-fidelity system environment incorporating full-wave electromagnetic modeling of a 3D biconical array and high-fidelity digital receiver simulation. The developed end-to-end modeling and evaluation framework provides a bridge between earlier theoretical studies of the DF method and future hardware prototyping and experimental validation.

**Abstract:**

A deep neural network-based 2D multi-octave omnidirectional direction-finding (DNN-based MOOD-DF) framework was previously developed using seven isotropic antenna elements arranged in a randomly distributed 3D array together with a simplified digital receiver model. In this paper, a system-level evaluation of the corresponding DF system is performed using a full-wave electromagnetic 3D DF array and high-fidelity receiver model. The isotropic elements and simplified receiver models are replaced with 2–18 GHz biconical antenna elements and high-fidelity digital receiver models that account for array feed structure effects, mutual coupling, receiver noise, cable length mismatches, and antenna receiver impedance mismatches within the RF receiving chain. Because full-array multi-octave electromagnetic simulations are computationally intensive, three representative frequency blocks (2–4 GHz, 10–12 GHz, and 16–18 GHz), spanning the lower, middle, and upper portions of the 2–18 GHz frequency range, are selected for DF system performance evaluation. The results demonstrate that the proposed DF system remains effective when trained and tested using the high-fidelity antenna array and receiver models. Across the investigated frequency bands, the system achieves sub-degree azimuth estimation accuracy and elevation RMSEs below approximately 1.8° at SNRs near 0 dB while maintaining microsecond-scale inference latency suitable for real-time applications. Although a performance gap relative to the Cramér–Rao lower bound remains, benchmark comparisons with the spectral MUSIC algorithm at selected frequencies indicate that the proposed approach can successfully operate on the complex and highly spatially aliased 3D manifold of the multi-octave biconical array, whereas conventional narrowband DF techniques may encounter substantial challenges under the same conditions. The presented end-to-end modeling and validation framework of the DF system provides a bridge between previous idealized studies and future hardware prototyping and experimental validation.

## 1. Introduction

In modern radar electronic support measures (RESMs) and radar electronic warfare (REW) systems, the ability to rapidly and accurately estimate the direction-of-arrival (DOA) or perform direction-finding (DF) on intercepted signals is essential for monitoring ultra-wideband radio-frequency (RF) activity in today’s congested and contested electromagnetic (EM) environment [1,2,3,4,5]. Consequently, DOA estimation for target tracking and localization continues to receive significant research attention. Over the past several decades, numerous approaches have been developed, including subspace-based methods [6,7] and maximum likelihood techniques [8,9]. Among these spectral subspace algorithms, most notably, multiple signal classification (MUSIC) [6] and estimation of signal parameters via rotational invariance techniques (ESPRIT) [7] remain the most established solutions, with a wide range of variants proposed in the literature [10,11,12,13,14,15,16,17].

In parallel with the above DOA estimation developments, recent research has expanded into compressive sensing (CS)-based methods [18,19,20,21], grid-less sparse recovery techniques [22], direct data-domain (DDD) processing [23,24,25,26], and innovative array configurations such as beam-scanning antenna architectures [27,28,29], to name just a few. CS-based DOA approaches exploit angular sparsity to achieve high resolution with fewer snapshots, while off-grid formulations mitigate discretization errors and enhance robustness in low-SNR environments. An overview of CS theory and its applications in wireless DF can be found in [30]. DDD methods have emerged as effective solutions for dynamic or non-stationary scenarios because they operate directly on raw sensor measurements without relying on explicit covariance estimation. By removing the requirement for long, stationary data records, these approaches maintain strong performance with short snapshots and naturally support spatiotemporal extensions and reduced-dimension beam space implementations, thereby improving agility and computational efficiency.

Despite their strengths and wide applications in RF signal DOA/DF estimation, these aforementioned methods often impose substantial computational burdens. For example, covariance-based techniques such as MUSIC require eigenvalue decomposition, CS approaches rely on ℓ_1_-regularization, and DDD processing involves matrix inversion. These demands hinder near-real-time deployment in REW environments, where multi-octave frequency bands must be monitored omnidirectionally in two dimensions, typically 360° in azimuth (az) and up to 100° in elevation (el). The challenge becomes even more pronounced under low-SNR conditions, when multiple signals may exceed detection thresholds simultaneously, necessitating ultrafast DF estimation and clustering strategies to suppress false alarms.

To address these challenges, recent research in DOA/DF estimation has pivoted toward data-driven paradigms. Deep neural networks (DNNs) and convolutional neural networks (CNNs) have emerged as powerful alternatives due to their ability to learn robust feature representations directly from raw data and/or preprocessed covariance matrices. For 1D DOA tasks, feedforward and convolutional architectures have been trained on raw complex snapshots or covariance features to predict the az angle [31,32]. In 2D scenarios, richer input representations, such as beam space images, multi-frequency steering vectors, and geometry-encoded tensors, enable joint az–el estimation [33,34]. Robustness to adverse environments has been enhanced through specialized designs: impulsive noise suppression via correntropy-weighted DNNs [35], coherent source separation using CNN-based spatial feature extraction [36], and wideband processing with learned time–frequency weighting [37]. Hardware and environmental mismatches are mitigated by training with simulated gain/phase errors, mutual coupling, and positional offsets [38,39], or by employing geometry-invariant encodings [40]. Emerging trends also integrate attention mechanisms [41] and complex-valued neural networks [33] to better exploit phase information in massive MIMO arrays.

Recent work also highlights a shift toward multi-task learning [42], jointly estimating both the number of sources and their DOAs, and toward transfer learning [43], where models trained on idealized simulations are adapted to real-world hardware data to compensate for array imperfections [44]. In addition, researchers are increasingly adopting complex-valued inputs [45,46] rather than magnitude-only representations, as preserving phase information is essential for accurate 2D DF. There is a growing trend toward hybrid DOA estimators that combine the spectral MUSIC subspace framework with DNNs, leveraging DNNs to learn array-manifold corrections, enhance covariance estimation, and improve robustness while preserving MUSIC’s super-resolution structure [47,48,49].

While most DNN-based 2D DOA methods focus on radar [50,51] and wireless applications [52,53,54], they often require prior knowledge of signal frequency and rely on regular-shaped arrays. To address multi-frequency 2D DOA, a frequency-unified DNN architecture with inhomogeneous angle-frequency partitioning was proposed in [55], using a non-uniform five-element array and a label-dependent loss function inspired by the Cramér–Rao lower bound (CRLB). However, the method was only demonstrated with a narrowband array in a low RF frequency band.

Unlike radar and wireless communication receivers, a distinguishing characteristic of RESM receivers is that the intercepted signal’s carrier frequency is unknown a priori. This necessitates a sequential processing chain in which frequency estimation precedes DOA/DF estimation. Given the 2–18 GHz monitoring span, RESM receivers must balance multi-octave coverage with fast, accurate DF processing. For example, ref. [56] reported that within a 12 ms observation window, more than 400,000 spectral peaks exceeded the detection threshold when multiple low-power FMCW radar signals were intercepted by a 6-element forward-looking DF array operating at extremely low SNRs. Here, “forward-looking” denotes DOA estimation restricted to a bounded angular sector, $\left[{az}_{1},{az}_{2}\right]\times \left[{el}_{1},{el}_{2}\right]$, typically covering a portion of the forward hemisphere relative to the platform’s orientation. Despite the large number of false alarms, DOA estimation was required for every detected peak, and K-means clustering was applied to suppress spurious responses. To mitigate this burden, ref. [57] introduced a multi-octave forward-looking DNN-based DF method that maps array steering vectors (ASVs) and estimated frequencies directly to az–el coordinates within the angular sector. The approach employs multiple DNNs, each using a uniform 16 × 1024 MLP architecture, achieving high accuracy and low latency across a wide frequency range, making it well suited for RESM and spectrum-monitoring applications requiring forward-sector DF.

Building on the forward-sector DF method introduced in [57], ref. [58] proposed a DNN-based 2D multi-octave omni-directional DF (DNN-based MOOD-DF) framework using an idealized 7-element isotropic 3D array with random element locations. That work demonstrated high DF accuracy together with single-digit-microsecond DNN inference time when performing batch inference on a GPU. However, like many recent DNN-based DOA/DF developments that rely on computer simulated data for training, validation, and testing [46,59,60,61], the data used in [58] to train and validate the models were generated using isotropic elements. As a result, mutual coupling within the 7-element 3D array and frequency-dependent radiation distortions in each element’s far field were not represented in model development. Therefore, further studies are necessary to incorporate realistic array effects and improve applicability to operational RESM systems.

To bridge the gap between theoretical studies and future hardware prototyping, from a DF system perspective, this work developed a 2–18 GHz biconical antenna element model based on full-wave electromagnetic (EM) simulations to replace the isotropic elements used in [58]. The resulting seven-element array was integrated with a seven-channel high-fidelity digital receiver model to generate normalized array steering vectors (ASVs) for a given frequency and direction. The DF system with DNN-based sub-band estimator was subsequently evaluated across selected frequency blocks within the 2–18 GHz operating band.

In order to achieve the primary objective of DF system evaluation, this work performs an end-to-end computational system-level evaluation of a multi-octave omnidirectional DF system using high-fidelity antenna array and receiver models. By integrating full-wave EM array modeling, high-fidelity digital receiver simulation, and DNN-enabled DF processing, the proposed DF system framework provides a bridge between previous idealized studies and future hardware prototyping and experimental validation. The main contributions are summarized as follows:

- An end-to-end DF system incorporating full-wave antenna array modeling and high-fidelity receiver simulation is developed. The results demonstrate that the proposed DF system remains effective in the presence of realistic antenna array effects, including mutual coupling, embedded-element pattern distortions, receiver noise, cable length mismatches, and antenna receiver impedance mismatches, while requiring no calibration tables during estimation.
- As summarized in Table 1, this work represents an important milestone in advancing the DF system from the idealized study reported in [58] toward future hardware prototyping and experimental validation.
- Across the investigated frequency bands, the DF system achieves sub-degree azimuth RMSE and elevation RMSE below approximately 1.8° at SNRs near 0 dB while maintaining microsecond-scale inference latency suitable for real-time applications.
- Benchmark comparisons with the CRLB and MUSIC provide theoretical and conventional performance references. The results show that the proposed DF system can successfully operate on the complex and highly spatially aliased 3D manifold of the multi-octave biconical array, whereas conventional narrowband DF techniques such as MUSIC may encounter substantial challenges under the same conditions.

The remainder of this paper is organized as follows. To develop a more realistic DF system model, Section 2 presents the design and performance of a 2–18 GHz biconical antenna element. In Section 3, the isotropic elements used in the theoretical study of the DNN-based DF framework are replaced with biconical elements, and the performance of the resulting seven-element 3D array is evaluated using full-wave EM modeling. Section 4 describes the receiver model from the embedded-element far-field patterns for a given direction and frequency to the normalized array steering vectors (ASVs). After establishing the full-wave array model and high-fidelity receiver model for the proposed DF system, Section 5 provides a brief overview of the previously developed DNN-based MOOD-DF framework employed in the system. Section 6 presents the trained DNN-based sub-band models for three representative frequency blocks (2–4 GHz, 10–12 GHz, and 16–18 GHz), which were selected to balance computational cost and modeling fidelity. Using these trained models, Section 7 evaluates the performance of the proposed DF system. Section 8 further assesses system performance through comparisons with the 3D array CRLB and the spectral MUSIC algorithm at representative frequencies. Section 9 discusses the implications and how to improve elevation estimation performance of the DF system. Finally, conclusions are drawn in Section 10.

Two points should be noted. First, to avoid ambiguity, the eight 2 GHz frequency blocks spanning 2–18 GHz used in [58] are hereafter referred to as frequency blocks, whereas the smaller frequency intervals within each block are referred to as sub-bands. Second, the SNR used throughout this paper is defined at the receiver input port and does not include any digital signal processing gain.

## 2. Full-Wave EM Modeling of a 2–18 GHz Metal Rod-Loaded Biconical Antenna

**Figure 1:**
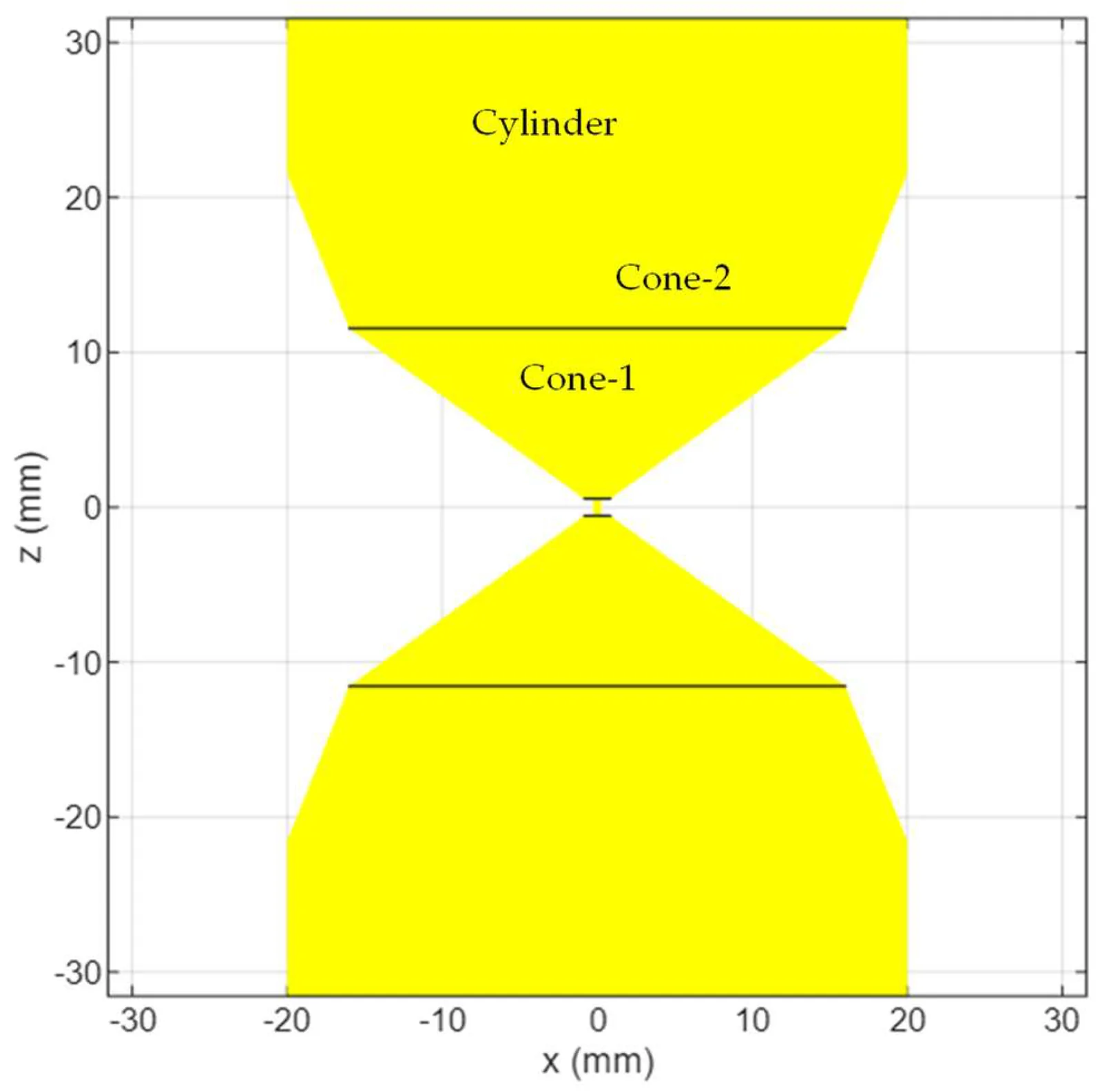
Side view of three-segment biconical antenna element.

Figure 1 illustrates the side view of the three-segment biconical antenna employed in this study. The geometry is mirror-symmetric about the XY plane and consists of two identical arms separated by a 1.1 mm feed gap. Each arm is composed of three sections: Cone 1, Cone 2, and a cylindrical termination. A discrete parameter search was performed to identify a geometry that satisfies the impedance bandwidth and omnidirectional radiation requirements across the 2–18 GHz operating band. Table 2 lists the dimensions of the antenna.

Following the modeling frameworks established in [62,63], the three-segment biconical antenna element was designed and modeled using the MATLAB (2025b) Antenna Toolbox. To achieve high-fidelity results, the element was excited via a cylindrical delta-gap feed positioned within the 1.1 mm gap between the two cones. The feed cylinder was specified with a diameter of 0.51 mm, deliberately matching the center pin diameter of a standard RG-405 rigid coaxial cable to ensure a realistic impedance transition.

In a physical array environment, elements are typically fed by rigid coaxial cabling, which can alter the radiating properties and local field distributions. To account for this, and drawing inspiration from the commercial implementation in [64], the model was augmented with a 100 mm long metallic rod with a 2.16 mm diameter. This metal rod serves to mimic the outer shell of the RG-405 rigid cable, transforming the idealized element into a metal “rod-loaded” configuration that more accurately captures the current distributions along the outside shell of coaxial feed line.

**Figure 2:**
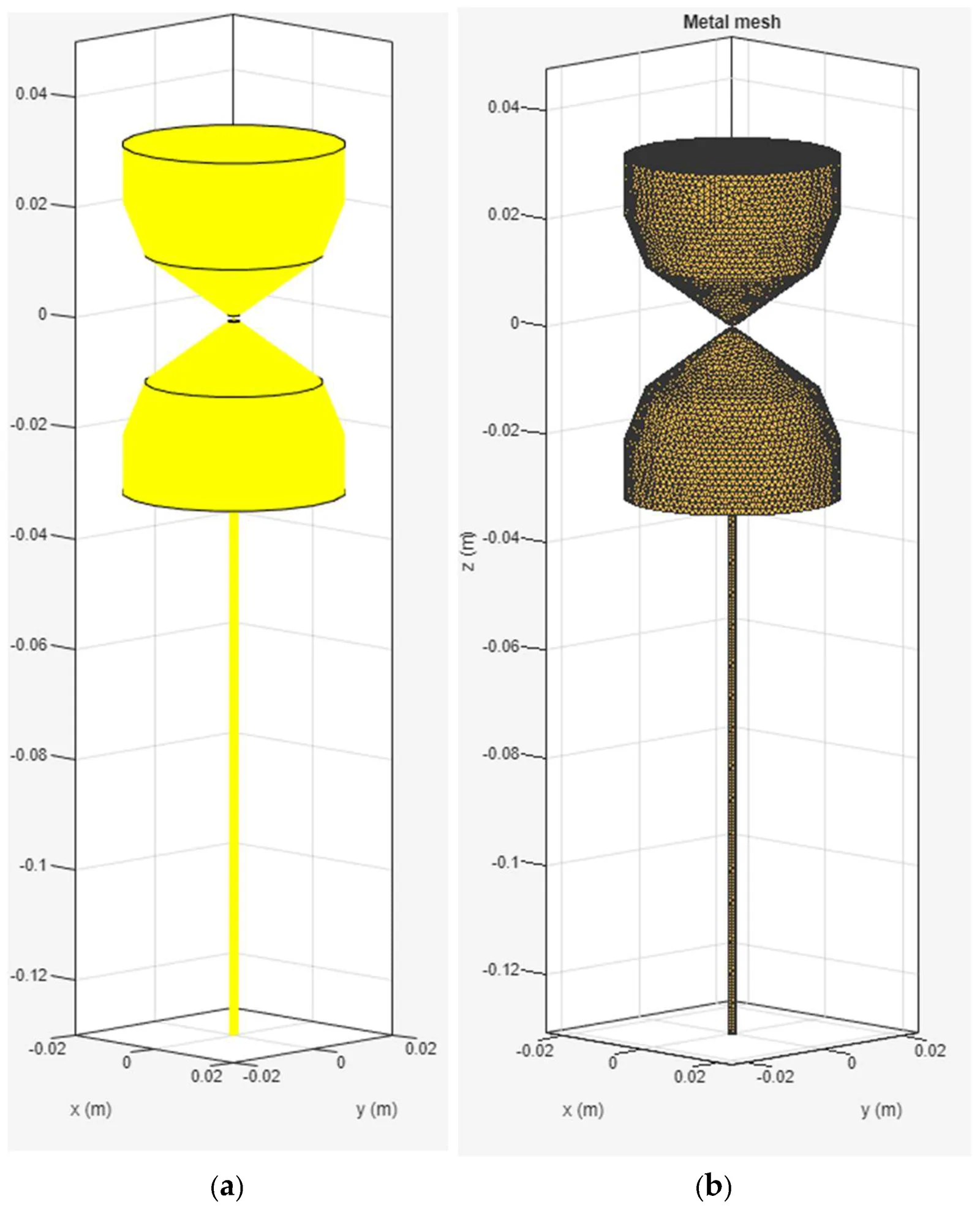
Geometry of the proposed antenna element: (**a**) the biconical structure loaded with a 100 mm metallic rod to emulate the coaxial feed shell, and (**b**) the corresponding discretized mesh utilized for the MoM full-wave EM analysis.

The rod-loaded element was analyzed using the Method of Moments (MoM) solver. Figure 2a illustrates the physical geometry of the biconical element and the cable shell, while Figure 2b depicts the corresponding triangular mesh generated for the full-wave EM solver.

**Figure 3:**
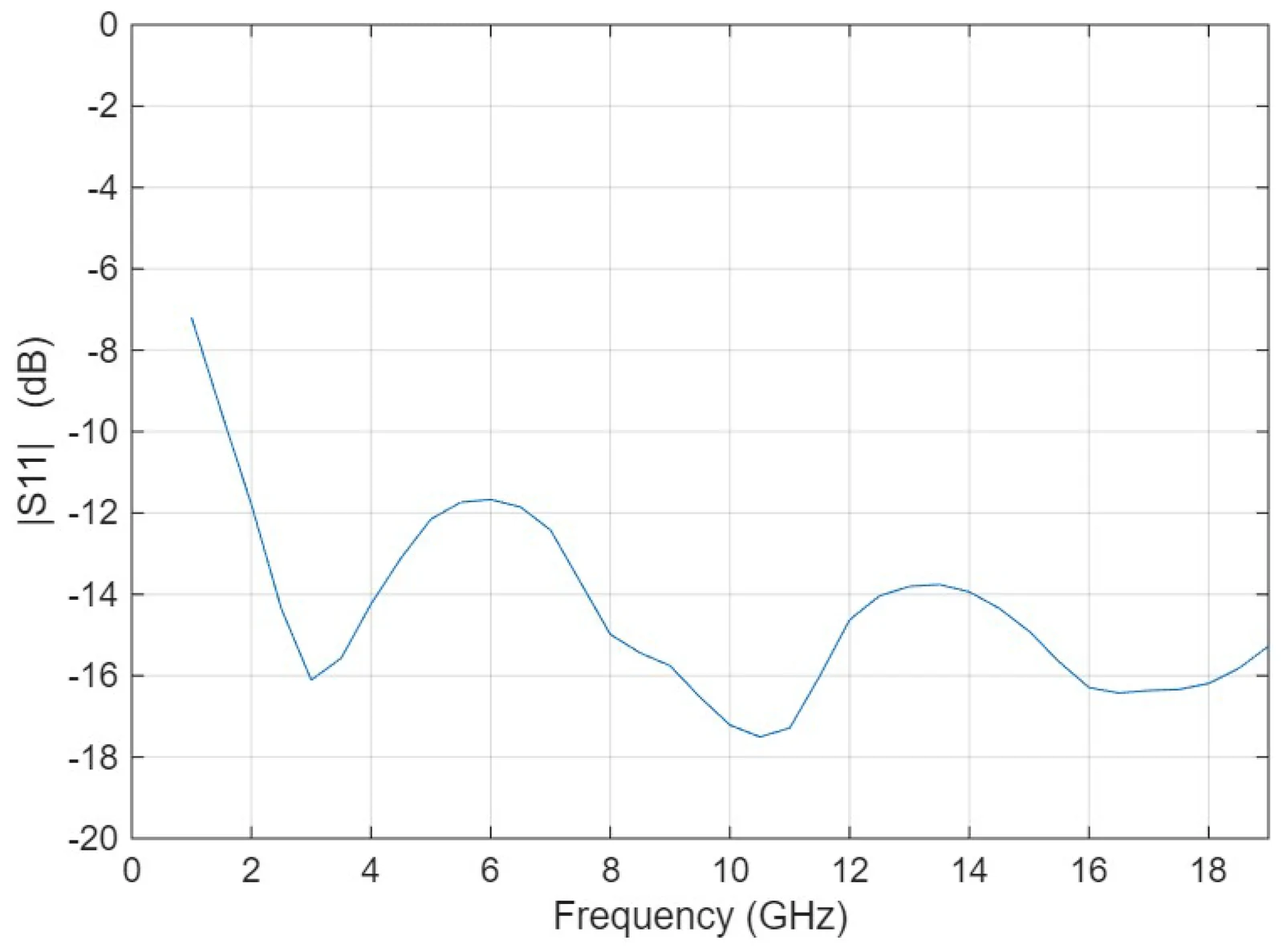
The computed S11 magnitude of the rod-loaded three-segment biconical antenna, demonstrating wideband impedance performance across 2–18 GHz.

To validate the element, the reflection coefficients were computed using the *sparameters* function in MATLAB. As shown in Figure 3, the results indicate that the rod-loaded element maintains a good impedance match to the 50 Ω feed across the desired bandwidth, confirming that the inclusion of the physical feed structure does not compromise the antenna’s wideband performance from an |S11| perspective.

Unlike conventional antenna studies that primarily focus on far-field radiation patterns and gains, this work emphasized the complex far-field E-field of the element. Based on the principle of antenna reciprocity, the calculated far-field E-field was used to determine the element vector effective length (VEL), detailed in Section 4, at a specified frequency and a given incident direction.

**Figure 4:**
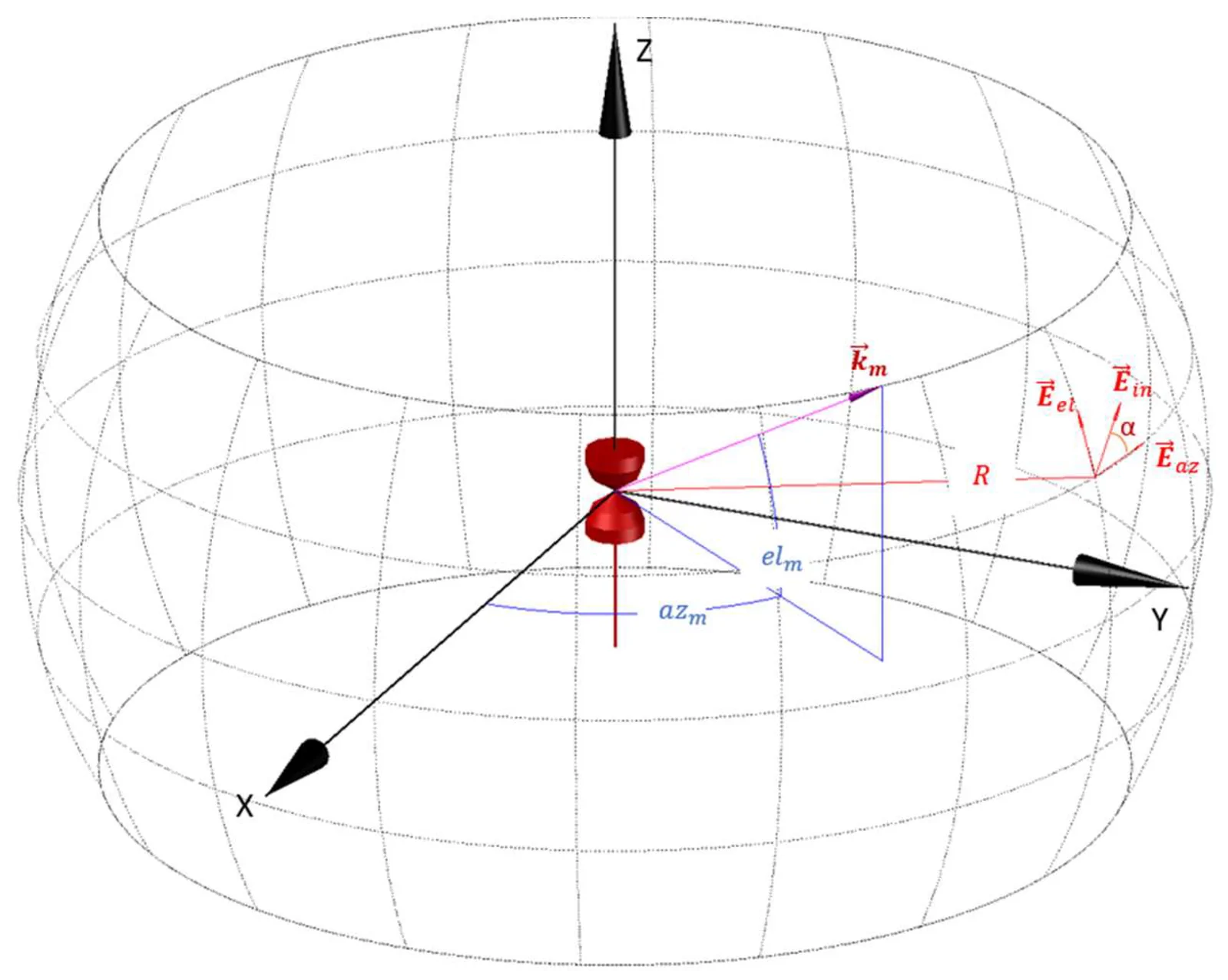
Antenna coordinate system and far-field local polarization coordinate system.

Figure 4 shows the element within its spherical coordinate system, where $\left({az}_{m},{el}_{m}\right)$ represents the signal direction. This direction can also be defined by the unit direction vector ${\hat{k}}_{m}$ = $\left[x_{m}\hat{x},{\;y}_{m}\hat{y},{\;z}_{m}\hat{z}\right]$*^T^*, where $\hat{x}$, $\hat{y}$ and $\hat{z}$ are unit vectors along the X, Y and Z axes, respectively. The projections of ${\hat{k}}_{m}$ onto these axes are given by the following:

(1) $$\{\begin{array}{c} x_{m}=\cos({el}_{m})\cos\left({az}_{m}\right)\\ y_{m}=\cos\left({el}_{m}\right)\sin\left({az}_{m}\right)\\ z_{m}=\sin\left({el}_{m}\right) \end{array}$$

The figure also depicts the local polarization coordinate system of an EM wave on a sphere of radius R. The three E-field components $\left({\vec{E}}_{az},{\vec{E}}_{el},{\vec{E}}_{r}\right)$ are mutually orthogonal and aligned with the $\left(\hat{az},\hat{el},\hat{r}\right)$ directions. Because the far-field of an antenna is normally characterized by a transverse electromagnetic (TEM) wave, the incident field ${\vec{E}}_{in}$ can be decomposed only into the transverse components ${\vec{E}}_{az}$ and ${\vec{E}}_{el}$; consequently, the radial component ${\vec{E}}_{r}$ is omitted from the figure. The polarization angle (α) is defined from the $\hat{az}$ direction toward $\hat{el}$ direction.

Based on the preceding discussion, the *EHfields* function from the MATLAB Antenna Toolbox was employed to compute the element E-fields at a distance of R = 100 m. Numerical simulations were performed across M=114,857 randomly sampled directions in the spherical coordinate system for 17 discrete frequency points spanning the 2–18 GHz range.

At each observation point, the function can yield three orthogonal E-field components $\left(E_{az},E_{el},E_{r}\right)$. The resulting 3D field distributions at the frequencies specified in Table 3 are provided in Appendix A. Table 3 further details the minimum ratios of $E_{el}/E_{az}$ and $E_{el}/E_{r}$. For those 3D field plots, el angles were limited within ±50°.

An analysis of these results indicates that the rod-loaded, three-segment biconical element operates as a linearly polarized element within the spherical coordinate system, with $E_{el}$ serving as the dominant field component. Consequently, the 3D far-field plots in Appendix A represent the $\left|E_{el}\right|$ component across the sampled directions. These plots clearly illustrate that the field distribution along the el direction exhibits ripples. This phenomenon indicates that the metallic rod functions as an integral part of the radiating element and breaks the symmetry of the biconical structure across the XY plane, thereby altering the symmetrical field distribution typically observed in the el direction.

## 3. Configuration of the Randomly Located 7-Element Biconical Array and Its Embedded-Element Far Fields

**Figure 5:**
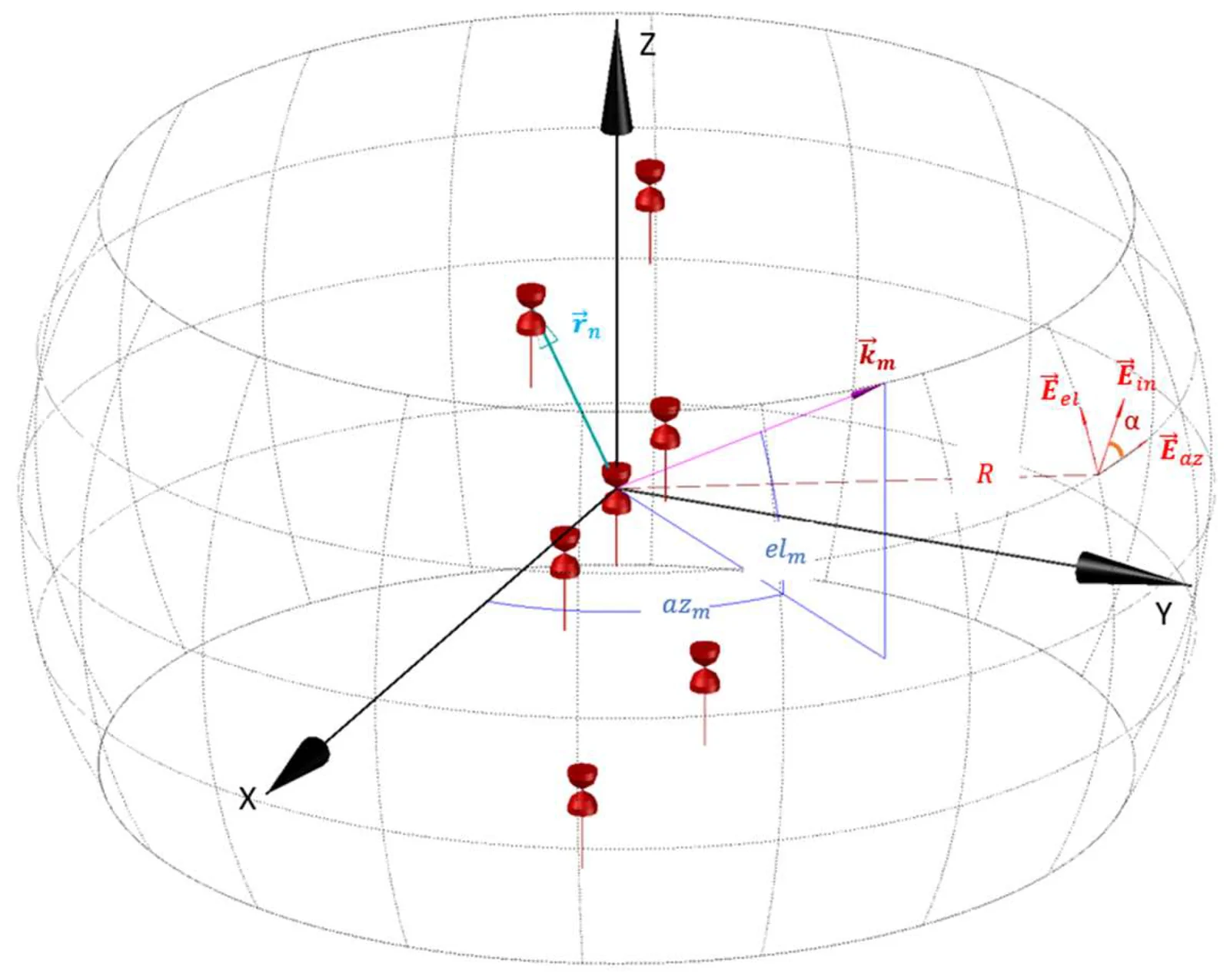
Randomly located 7-element biconical array and coordinate system.

Using isotropic element locations studied in [58], shown in Table 4, and the rod-loaded three-segment biconical antenna, the 7-element DF array is illustrated in Figure 5. The reasons for these element locations were also discussed in [58].

In the figure, the first (reference) element is at the origin of Cartesian coordinates. The vector ${\vec{r}}_{n}$ is the displacement from the phase center of the reference element to the phase center of the $n^{th}$ element in the array (n=1, 2, 3,…N=7), and the unit direction vector ${\hat{k}}_{m}$ indicates the $m^{th}$ signal direction.

**Figure 6:**
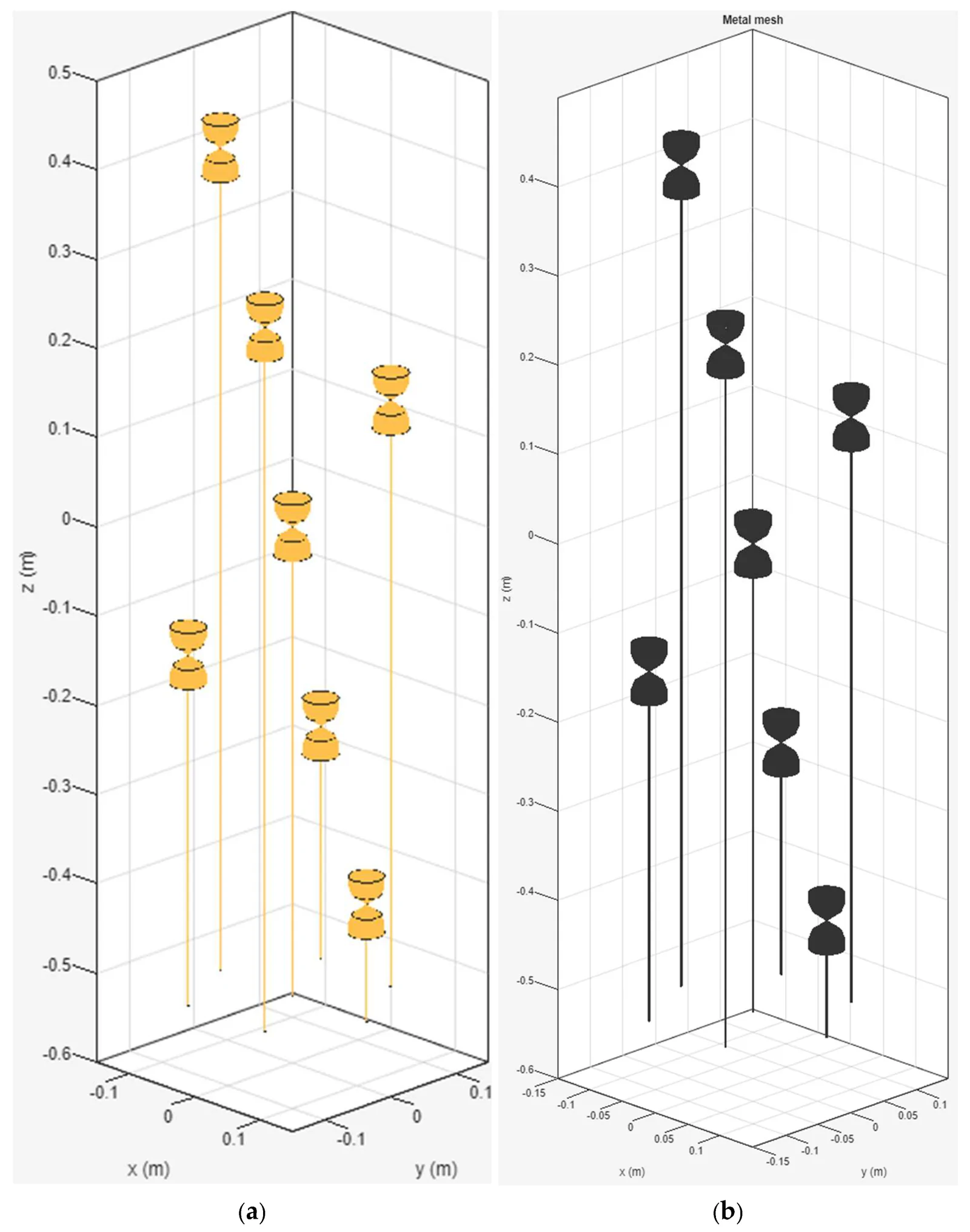
(**a**) The geometry of the 7-element biconical array, and (**b**) the corresponding discretized mesh utilized for the MoM analysis.

The complete array geometry, modeled in the MATLAB Antenna Toolbox, is illustrated in Figure 6a. Because the elements were randomly distributed in 3D space, the lengths of the RG-405 rigid coaxial feed lines varied accordingly, as shown in the figure. It is assumed that the array is fed from the bottom.

The corresponding meshed geometry is presented in Figure 6b. For the MoM calculations, the mesh consists of 100,398 triangular elements. The MoM simulation accounts for all mutual EM coupling between the biconical elements, including the parasitic EM effects introduced by the RG-405 rigid coaxial feed lines.

**Figure 7:**
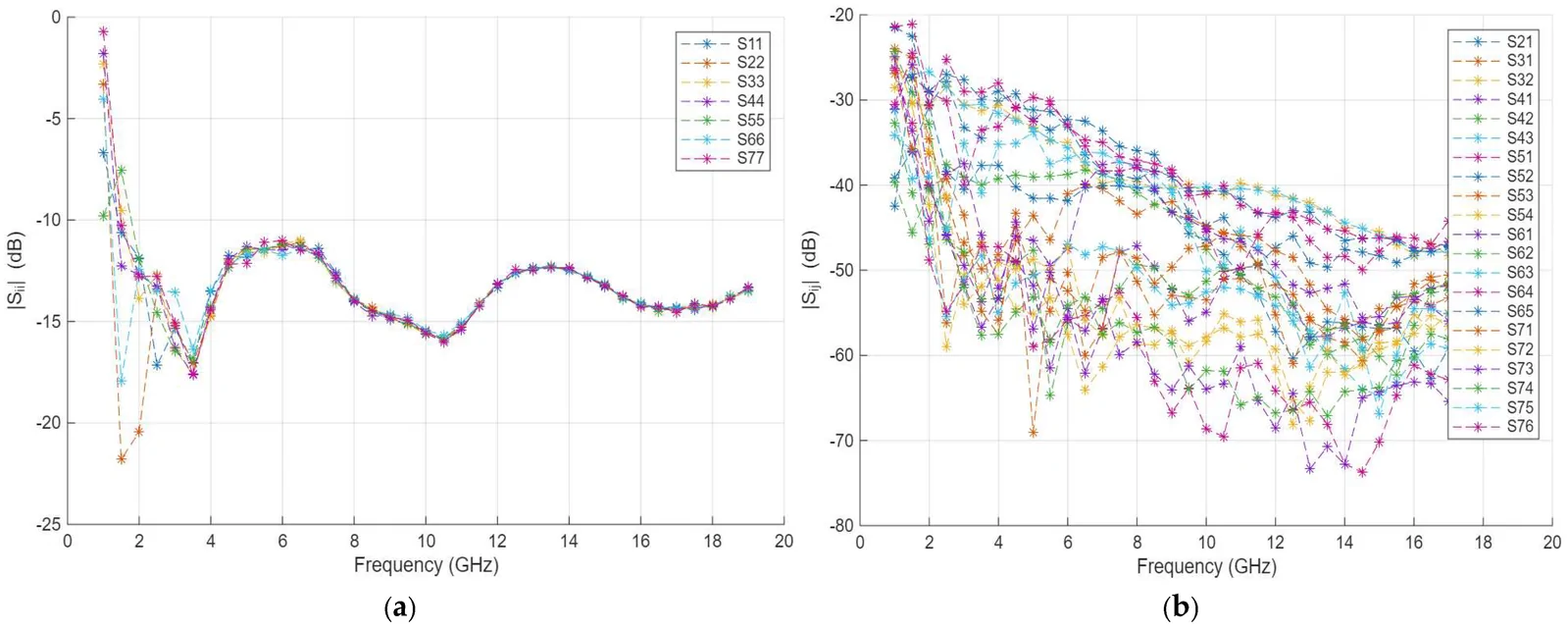
Full-wave EM calculated S-parameter results for the 7-element array across 2–18 GHz: (**a**) $S_{ii},(i=1,\;2,\ldots 7)$ and (**b**) $S_{ij},(i,j=1,\;2,\ldots 7)$.

The 7-element array S-parameters are shown in Figure 7. As seen in Figure 7a, all elements exhibited good impedance matching across the entire operating band, with input reflection coefficients $\left(S_{ii}\right)$ remaining below −10 dB. Figure 7b shows that the mutual coupling $\left(S_{ij}\right)$ between elements stayed below −22 dB from 2 to 18 GHz, indicating very low interaction among elements. The coupling levels decreased further with increasing frequency, a behavior attributed to the larger inter-element spacing in terms of wavelengths. These low coupling levels were maintained across the band despite the presence of the metallic feed rods. This performance arises from a well-balanced 3D element arrangement. A strictly collinear vertical stack reduces mutual coupling by exploiting the nulls of the biconical element ‘donut’ radiation pattern, but it provides insufficient az resolution. Conversely, excessive spreading element locations in the XY plane may increase coupling. The adopted moderately dispersed 3D geometry preserves high inter-element isolation while maintaining the az aperture required for omnidirectional DF.

The embedded-element far fields on a 100 m radius sphere were computed and some examples are provided in Appendix B. Instead of presenting all seven elements’ full 3D embedded element far-field distributions at the 17 frequency points spanning 2–18 GHz, Table 5 lists the embedded far-field distributions of selected elements at specific frequencies referenced in Appendix B. In this work, an element’s embedded far field refers to the radiation pattern obtained when that element was excited within the array while all other elements were terminated with 50 Ω loads during the MoM calculations.

As illustrated by the three-dimensional far-field distributions in Appendix B, the embedded-element radiation patterns exhibit increasingly pronounced ripple structures at higher frequencies over the surface of the 100 m radius observation sphere. These distortions result from electromagnetic interactions among the array elements, including mutual coupling and scattering from the metal rods. As frequency increases, the embedded-element patterns become progressively more complex, exhibiting a larger number of local maxima, deeper nulls, and faster angular variations. These characteristics increase the complexity of the array manifold and are later shown in Section 8 to have a significant impact on the performance of spectral MUSIC-based DF.

Table 5 also summarizes the minimum ratios of ${|E}_{el}/E_{az}|$ and ${|E}_{el}/E_{r}|$ for each embedded far-field distribution shown in Appendix B. For each frequency point, one array element was randomly selected, and no specific criterion was applied when choosing these frequency–element pairs. Consequently, the ratios reported in Table 3 and Table 5 are not directly comparable on a one-to-one basis, even though the same set of frequency points was used. Nevertheless, a general comparison of the minimum |$E_{el}/E_{az}|$ values in both tables indicates that the element’s linear polarization is reduced in the DF array environment due to array-induced electromagnetic interactions.

**Figure 8:**
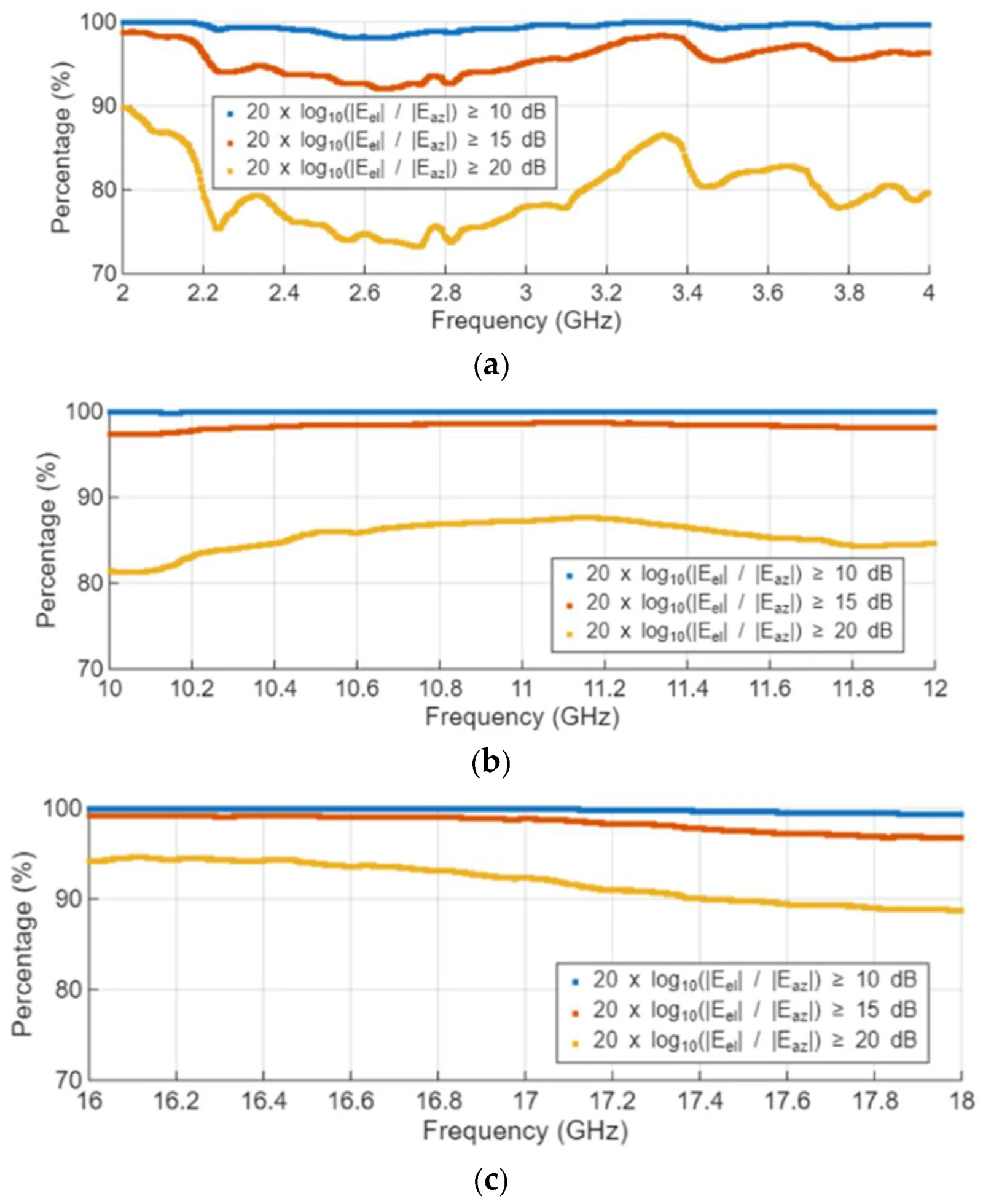
The percentage of 114,857 directions where all 7-embedded-element far fields meet the condition $20\times \log_{10}\left(\left|E_{el}\right|/\left|E_{az}\right|\right)$ ≥ 10, 15 and 20 dB versus frequency for (**a**) 2–4 GHz, (**b**) 10–12 GHz, and (**c**) 16–18 GHz, respectively.

Figure 8 shows percentages of M directions where all of the 7-embedded-element far fields meet $|E_{el}|/{|E}_{az}|\geq$ 10, 15 and 20 dB. It can be observed that more than 92% of directions satisfy the 15 dB criterion, and this percentage increases to more than 96% as frequency increases.

Despite the overall $\left|E_{el}/E_{az}\right|$ reduction from a single element to the array environment, the results in Table 5 and Figure 8 confirm that the embedded radiation patterns of the 7-element biconical array maintain good linear polarization along the el direction across the three bands (2–4, 10–12, and 16–18 GHz) used to demonstrate the proposed approach. Therefore, without loss of generality, only $E_{el}$ polarization is considered in the remainder of this paper to demonstrate the proposed DF data generation methodology. Another reason for using $E_{el}$ for the DF array is that the final system will include a radome with an integrated polarizer [65,66]. As a result, the DF array inside the radome will predominantly receive signal from the $E_{el}$ component.

## 4. DF System Model: From Embedded-Element Far Fields to Measured ASVs

To generate training and testing datasets for the DNN-based MOOD-DF model, this section describes the transition from simulated embedded-element far fields to synthetic ASVs generated using full-wave EM modeling and receiver simulation. The procedure incorporated the physical effects of mutual coupling, impedance mismatch, and receiver noise for a 7-element biconical array and a multi-channel digital receiver. The resulting ASVs reflected the complex voltages delivered to each receiver channel after Fast Fourier Transform (FFT) processing.

### 4.1. Reciprocity and Vector Effective Length (VEL)

Under Lorentz reciprocity, assuming a linear, time-invariant, reciprocal medium, the embedded-element far-field patterns inherently include all EM mutual coupling and scattering effects. The embedded elements’ far fields were obtained by exciting one element at a time while terminating all other six ports with 50 Ω loads. When normalized to a unit excitation current $I_{in}(n)$, the embedded-element far fields define the complex VEL [67], ${\vec{h}}_{eff}\left(f,m,n\right)$, for frequency f, direction m, and channel index n. In the spherical basis $\left(\hat{az},\hat{\;el}\right)$, the el component of the VEL is as follows:

(2) $$h_{eff,el}\left(f,m,n\right)=\frac{-j4\pi Re^{jkR}}{\eta_{0}{kI}_{in}(n)}\left[E_{el}\left(f,m,n\right)\right],$$

where R is the far-field radius, $\eta_{0}$ is the free-space impedance, and k=2π/λ and λ is the free-space wavelength. Based on the reasons discussed in Section 3, only the $E_{el}$ component was used in DNN model development. Using the VEL, the induced open-circuit voltage at the $n^{th}$ port for an incident plane wave ${\vec{E}}_{in}(f,m,n)$ from direction m is as follows:

(3) $$V_{oc}\left(f,m,n\right)={\vec{h}}_{eff,el}\left(f,m,n\right)\cdot {\vec{E}}_{in}\left(f,m,n\right).$$

### 4.2. Receiver Chain Transfer Function

The antenna open-circuit voltage cannot be directly sampled by the digitizers and must propagate through the antenna–receiver interface. Based on Thévenin/Norton equivalent circuit theory and microwave transmission line impedance transformation [68], a complex voltage transfer function H(f,n) is defined in (4) to relate the antenna open-circuit voltage to the receiver input voltage:

(4) $$H\left(f,n\right)=\frac{\left(1-\Gamma_{A}\left(f,n\right)\right)\left(1+\Gamma_{R}\left(f,n\right)\right)e^{-j\beta \left(f,n\right)l(n)}}{2[1-\Gamma_{A}\left(f,n\right)\Gamma_{R}\left(f,n\right)e^{-2j\beta \left(f,n\right)l\left(n\right)}]}$$

The transfer function accounts for the antenna embedded reflection coefficient $\Gamma_{A}(f,n),$ the receiver input reflection coefficient $\Gamma_{R}(f,n)$, and a lossless interconnecting cable of length l(n) with propagation constant β(f,n). Importantly, while the open-circuit voltage depends on the spatial arrival angles, this guided-wave transfer function remains strictly independent of the EM wave incident direction.

The delivered voltage phasor at the $n^{th}$ channel for direction m is as follows:

(5) $$\left|V_{del}(f,m,n)\right|e^{j\varphi (f,m,n)}=V_{oc}\left(f,m,n\right)H\left(f,n\right)$$

It was assumed that a MACOM broadband low-noise amplifier (LNA), model MAAL-011130 [69], served as the first-stage amplifier in each receiver channel. The measured S-parameters of the LNA, supplied in Touchstone (.*s*2*p*) format, were directly incorporated into the simulation. Identical S-parameter data of the LNA were used for all seven receiver channels.

### 4.3. Signal Synthesis and ASV Extraction

The simulation emulated the full receiver chain from RF to intermediate frequency (IF). The analytic RF signal at channel n for direction m is expressed as follows:

(6) $$v\left(t,m,n\right)=\left|V_{del}\left(f,m,n\right)\right|e^{j(2\pi ft+\varphi \left(f,m,n\right))}$$

Down-conversion with a local oscillator at $f_{LO}$ produces an IF signal as follows:

(7) $$v_{IF}\left(t,m,n\right)=v\left(t,m,n\right)e^{-j(2\pi f_{LO}t)}$$

The IF signal was sampled at $f_{s}$, exceeding the Nyquist rate. The noise power was computed from the RF receiver noise figure (NF) and thermal noise floor $k_{B}TB$ ($k_{B}$: Boltzmann’s constant; T: absolute temperature in kelvin; and B: noise bandwidth in hertz). Complex additive white Gaussian noise, w(i,n), was introduced to yield the sampled signal:

(8) $$r_{IF}\left(i,m,n\right)=v_{IF}\left(i,m,n\right)+w\left(i,n\right),\;\;\;\;\;i=1,2,\ldots$$

where i denotes the time-domain sample index.

A P-point FFT was applied to the sample data in (8). Let V(b,m,n) denote the FFT coefficient at bin b. The bin $b^{*}$ corresponding to the spectral peak in the reference channel was identified, and the calculated ASV was formed as

(9) $$ASV\left(f_{b^{*}},m\right)={\left[\begin{array}{ccc} 1 & \frac{V(b^{*},m,2)}{V(b^{*},m,1)} & \begin{array}{ccc} \frac{V(b^{*},m,3)}{V(b^{*},m,1)} & \ldots & \frac{V(b^{*},m,7)}{V(b^{*},m,1)} \end{array} \end{array}\right]}^{T},$$

where $f_{b^{*}}$ is the estimated frequency after FFT.

By directly incorporating hardware-induced phase and amplitude variances, such as channel-specific cable lengths, multiple internal reflections between the antenna and receiver, and varying receiver noise figures, along with EM coupling into the synthetic training datasets, the DNN model learns a robust mapping that implicitly compensates for system non-idealities. This data-driven approach to DF model development provides two major engineering advantages: it relaxes strict hardware tolerances during manufacturing, and inference does not consult a separate amplitude/phase calibration table, because the impairments are already included in the training ASVs.

### 4.4. Mimicking Chamber Data Collection for a Specified SNR Level in the Receiver

In chamber measurements, transmit power is always adjusted to achieve a target SNR in the measured data once the transmitting and receiving antenna geometry and receiver noise figures are fixed. In simulation, this was implemented by identifying the incident field strength that produced the desired SNR, hereafter referred to as the targeted SNR. The required open-circuit voltages $V_{oc}\left(f,m,n\right)$ were computed by inverting the receiver chain logic discussed above. Because VEL varies with m and n at a specified frequency, an average incident field magnitude was defined:

(10) $$E_{in,avg}\left(f\right)=\frac{1}{MN}\sum_{m}\sum_{n}\frac{V_{oc_{req}}\left(f,m,n\right)}{\left|{\vec{h}}_{eff,el}\left(f,m,n\right)\right|}$$

Using $E_{in,avg}\left(f\right)$ as the nominal incident field produced a distribution of SNR values across channels, due to the non-uniform spatial behavior of $h_{eff,el}\left(f,m,n\right)$.

## 5. Brief on DNN-Based MOOD-DF Model

While the theoretical foundation of the DNN-based MOOD-DF framework has been detailed in [58], this section provides a self-contained summary of the method’s architecture and feature mapping. The primary objective of the DNN-based model is to map the high-fidelity ASV data and frequency information directly to the signal’s arrival unit direction vector in Cartesian coordinates.

### 5.1. Input Feature Vector and Normalization

To ensure that the DNN learns effectively from the multi-octave data generated in Section 4, all input features are normalized to a common range of [−1, 1].

- ASV Features: The complex-valued ASV from (9) was decomposed into its real and imaginary components. By referencing to the first element, an N-element array produces 2(N−1) real-valued features.
- Frequency Mapping: Training a single DNN model to cover multiple octaves would increase the difficulty of training and inference complexity, so the multiple sub-band strategy was introduced [58]. Using mapped frequency as part of the DNN model input feature effectively eliminates frequency ambiguity, which commonly arises in ultra-wideband arrays. For the $S^{th}$ sub-band, its frequency is linearly mapped to the range [−1, 1]:
  (11) $$f_{map}=-1+\frac{2*\left(f_{b^{*}}-f_{1}\left(S\right)\right)}{f_{2}\left(S\right)-f_{1}\left(S\right)}$$
  where $f_{1}(S)$ and $f_{2}\left(S\right)$ are the sub-band boundaries. This prevents the large numerical values of the frequency from dominating the gradient updates during DNN training.

### 5.2. Multi-Layer Perceptron (MLP) Architecture

**Figure 9:**
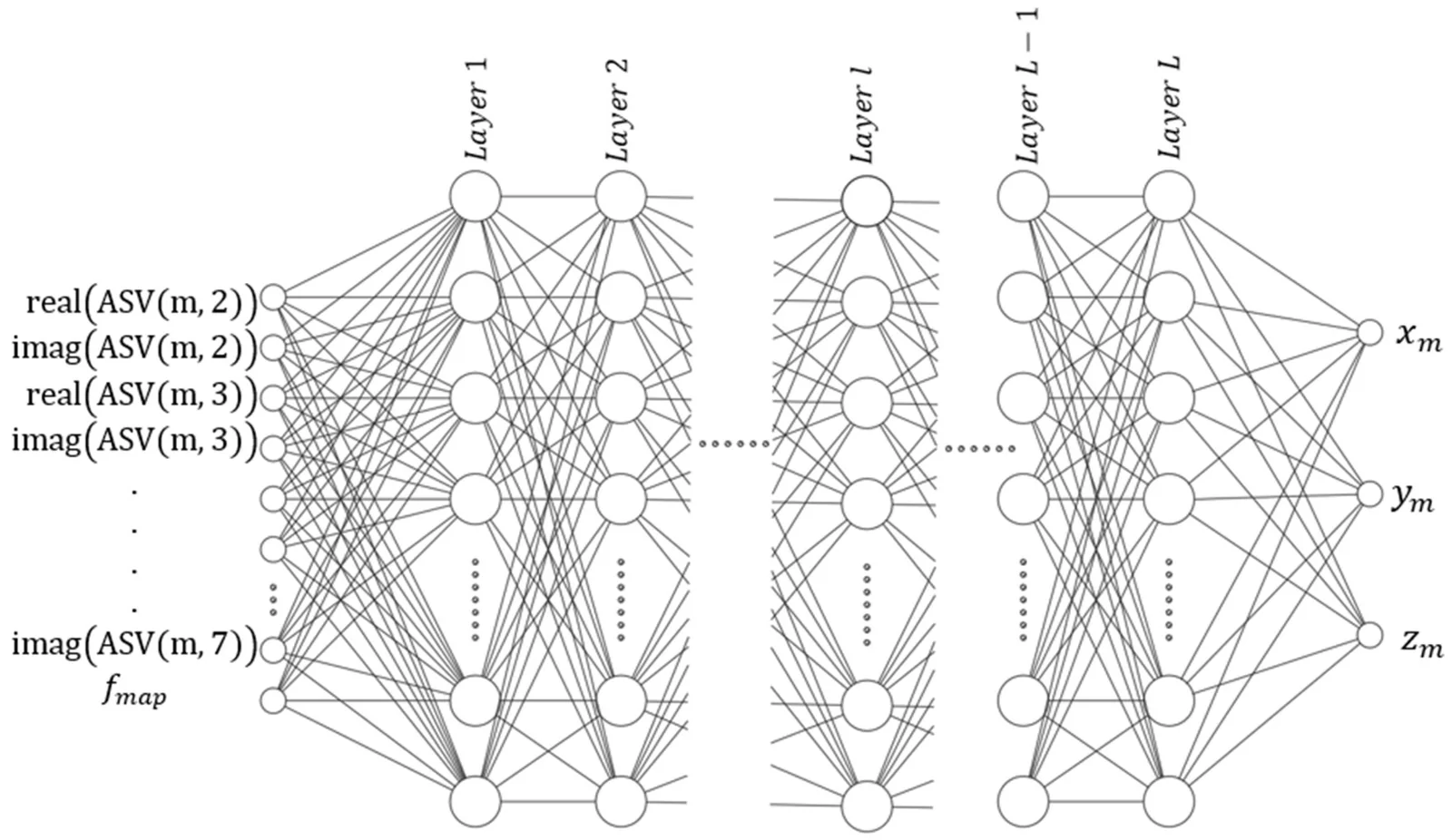
Block diagram of DNN-based DF-model with L uniform MLP layers. Redrawn and adapted from [58]. © 2026 Crown Copyright.

In this study, the DNN-based MOOD-DF model employs an MLP architecture, as illustrated in Figure 9. For each sub-band, the network consists of the following:

- Input layer: 1+2(N−1) neurons (13 neurons for a 7-element array).
- Hidden layers: A uniform structure of L layers, each containing $h_{l}$ neurons with a non-linear activation function, (e.g., ReLU).
- Output layer: Three outputs representing the predicted Cartesian unit direction vector defined in (1).

Using the ASVs from Section 4, the MLP learns the array-specific coupling and hardware differences through its weights. Those differences are not measured as a separate calibration product and are not removed from the ASV before inference.

The resulting sample vector for DNN model training, validation and testing is defined as follows:

(12) $$sample=\left[\begin{array}{c} \begin{array}{c} real(ASV\left(m,2\right))\\ imag(ASV\left(m,2\right))\\ \begin{array}{c} real(ASV\left(m,3\right))\\ imag(ASV\left(m,3\right)) \end{array} \end{array}\\ \begin{array}{c} \vdots \\ real(ASV\left(m,7\right))\\ \begin{array}{c} \begin{array}{c} imag(ASV\left(m,7\right))\\ f_{map} \end{array}\\ \begin{array}{c} x_{m}\\ \begin{array}{c} y_{m}\\ z_{m} \end{array} \end{array} \end{array} \end{array} \end{array}\right]$$

## 6. DF System Evaluation in Three Representative Frequency Blocks

This section details the methodology used to evaluate the proposed DF system, including full-wave EM data generation, digital receiver simulation, and the development of DNN-enabled DF estimators within representative frequency blocks.

### 6.1. Data Sample Generation

To demonstrate the DF method, the 2–18 GHz band was divided into eight 2 GHz frequency blocks in [58] using isotropic elements, and a separate DNN model was trained in each block.

As noted in Section 1, the full-wave EM computations of every element’s embedded-element far fields of the 7-element DF array were extremely time-consuming when evaluated over vast random directions and dense random frequency samples, e.g., more than 114,000 directions and 1256 frequencies within a 2 GHz frequency block.

During sample generation, full-array MoM calculations were performed on a dedicated Dell Precision 7960 workstation equipped with an Intel Xeon W9-3475X processor (36 cores, 72 threads) and 383 GB of RAM. Using the MATLAB Parallel Computing Toolbox, 14 parallel workers were employed due to RAM limitations (despite more cores being available), since each frequency point required approximately 25 GB of memory and an average of 8 h to compute the far-field responses over all directions. Consequently, generating the dataset for a single 2 GHz frequency block over the specified angular-frequency grid required approximately one month of continuous computation.

Given the substantial computational cost of the full-wave simulations, three representative frequency blocks (2–4 GHz, 10–12 GHz, and 16–18 GHz) were selected from the eight frequency blocks considered in [58]. These blocks correspond to the lower, middle, and upper portions of the 2–18 GHz operating band and therefore provide representative coverage of the frequency range of interest. Furthermore, the DF array exhibits good input matching and consistently low mutual coupling across the full band, while the same receiver model is employed throughout the data generation process. Combined with the full-band results reported in [58], these representative cases provide a physics-based assessment of the proposed approach using a full-wave EM array model. The remaining frequency blocks can be analyzed using the same EM modeling, data generation, and DNN training procedure, and full-band tests will be reported in subsequent experimental papers.

A key advantage of the DNN-based MOOD-DF method is that sub-band sizes within a given frequency range can be chosen arbitrarily, since the estimated frequency from the RESM receiver is included as part of the DNN input feature vector (12). In this study, different sub-band sizes smaller than the original 2 GHz blocks were evaluated within these three selected frequency blocks.

Table 6 lists the parameters used in the digital receiver model to generate samples in (12) in each sub-band to train, validate and test the corresponding DNN model.

To generate directions that are uniform with respect to surface area on the unit sphere, each sample was drawn as a three-dimensional vector with *i.i.d.* standard-normal components and then normalized to unit length (Muller’s method). Because the isotropic Gaussian is rotationally invariant, the resulting points are uniformly distributed on the sphere, and every area element has equal probability. This avoids the polar crowding that occurs if az and el are sampled uniformly (equiangular spherical-coordinate sampling). Samples falling outside the prescribed angular region were discarded; so, the retained directions remain uniform on that spherical subset.

**Figure 10:**
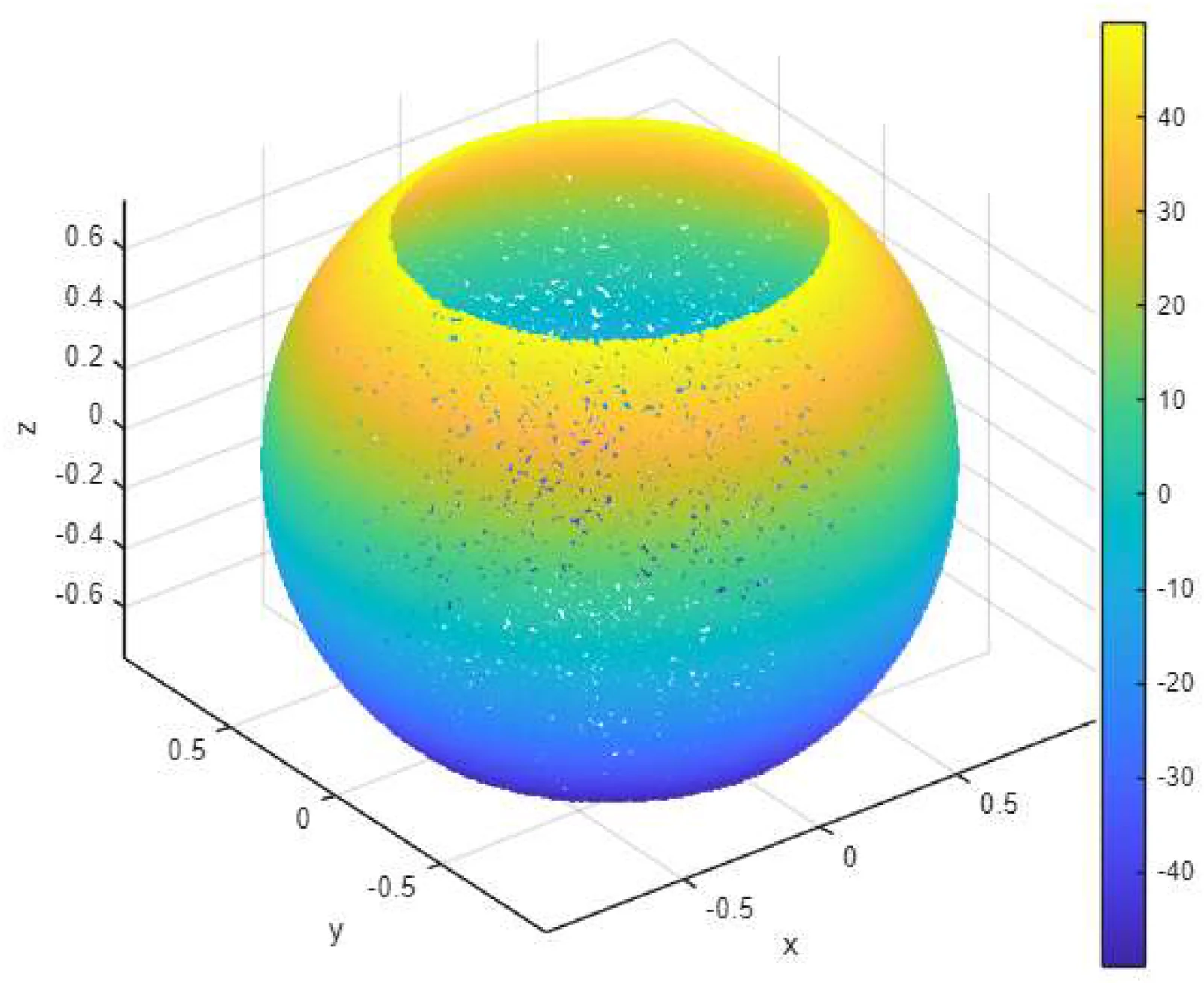
An example of uniformly distributed directions on a unit sphere via Gaussian normalization with an el limit of ±50°.

**Figure 11:**
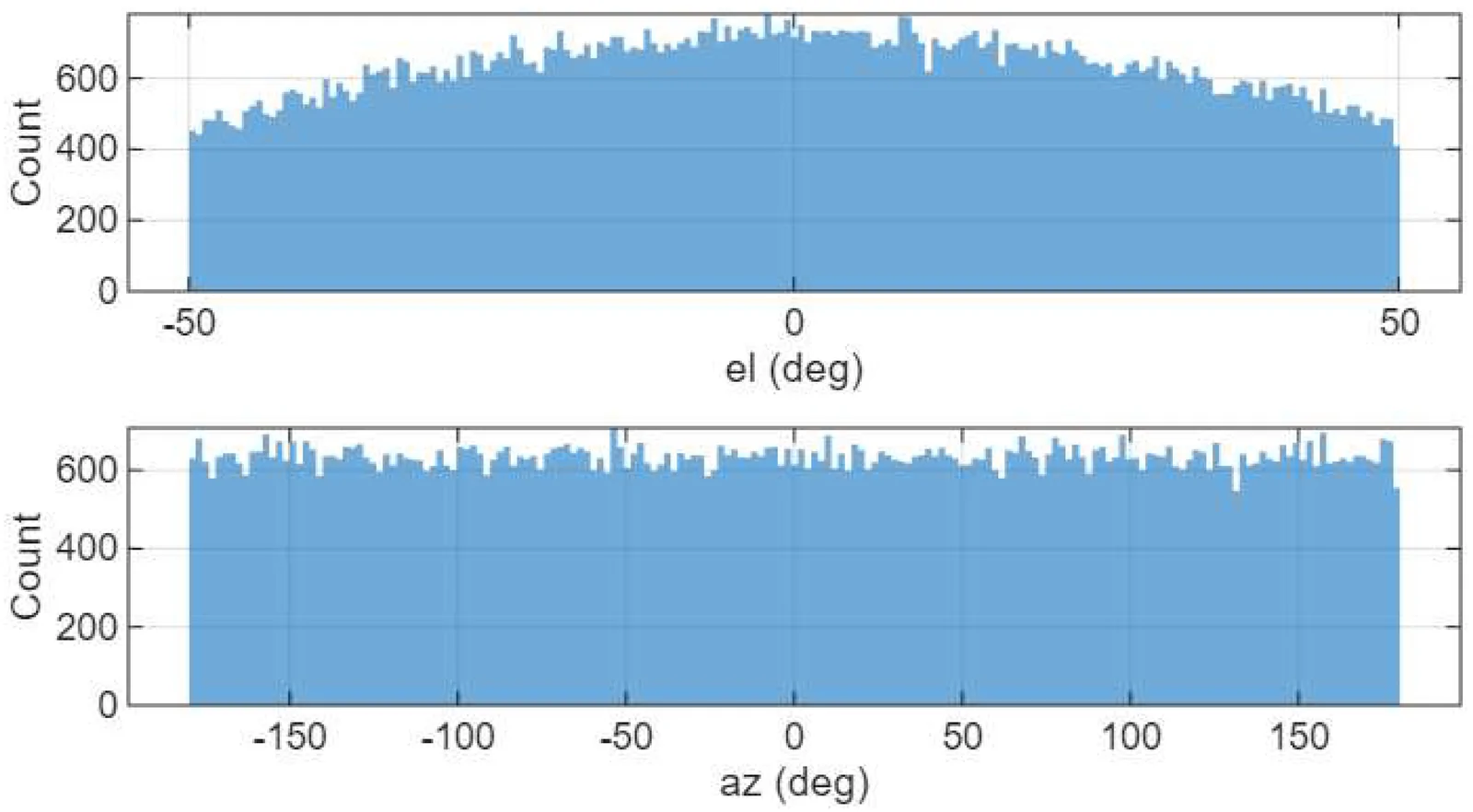
az and el distributions of uniformly distributed directions on a unit sphere with an el limit of ±50°, as shown in Figure 10.

Figure 10 shows example retained sets. Figure 11 shows the corresponding az and el histograms; az is essentially uniform, while el follows a cos(el) weighting, as required for an area-uniform spherical sample. These directions were used for the embedded-element far-field calculations.

**Figure 12:**
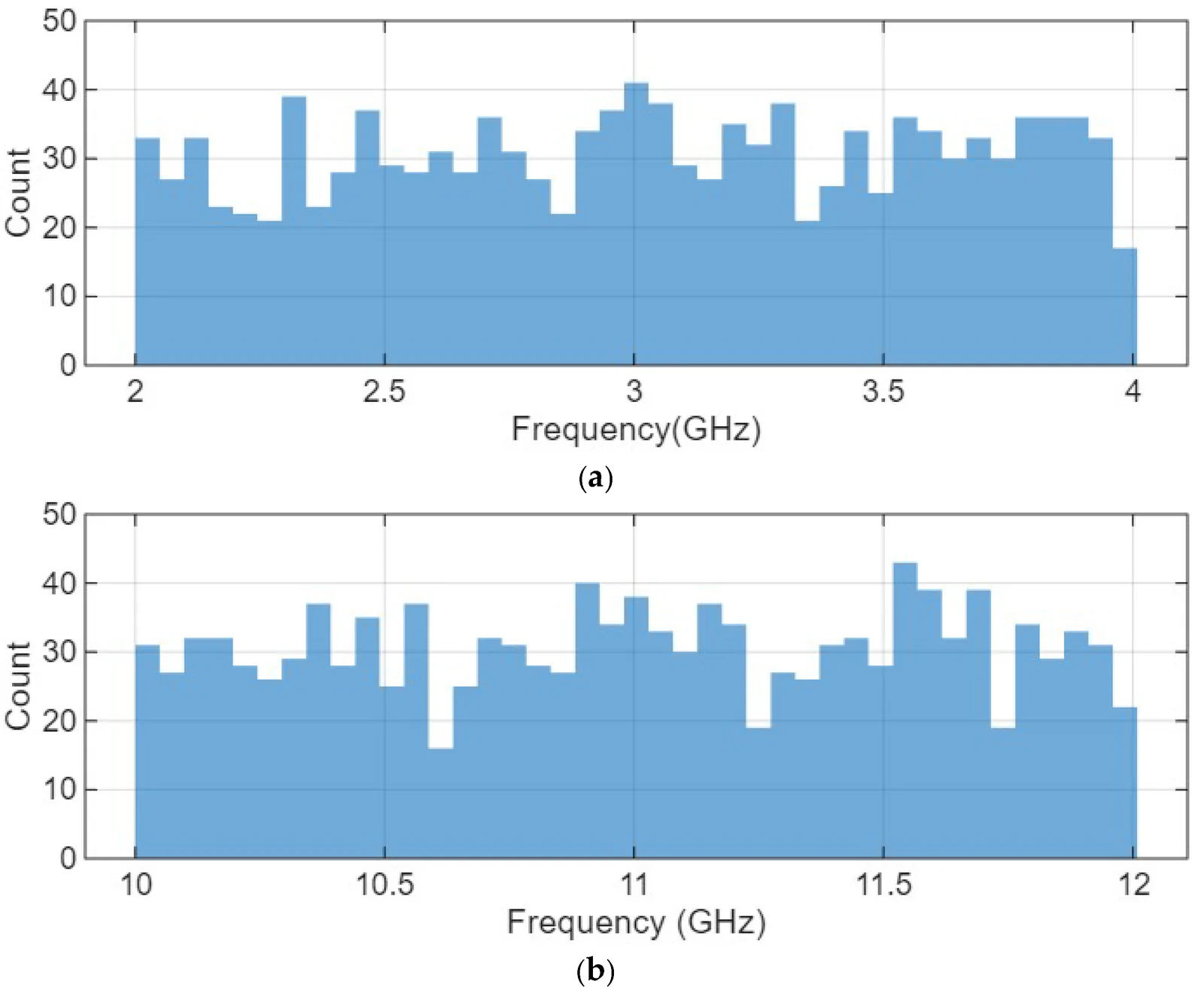

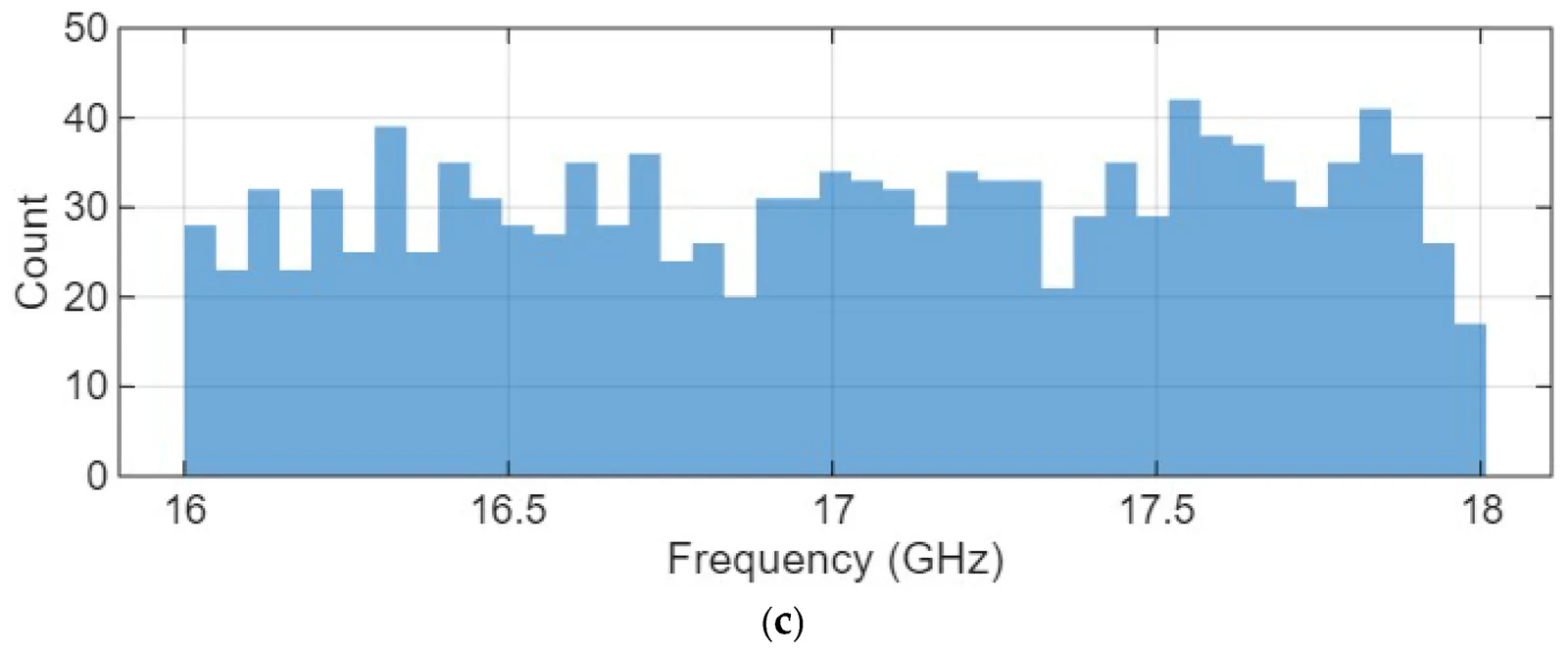
Randomly selected frequency distributions across the three studied frequency blocks: (**a**) 2–4 GHz, (**b**) 10–12 GHz, and (**c**) 16–18 GHz.

Figure 12 illustrates the randomly selected frequency distributions across the three studied bands, with each band containing 1256 frequency points. For each frequency, M=114,857 directions were utilized for the embedded-element far-field calculations when the el limit was ±50°. The resulting far-field data were subsequently employed to construct the ASV(f,m).

Using an el limit of ±50° as the example, there were 144,260,392 samples defined in (12) generated in each 2 GHz range used to train, validate and test the DNN models.

### 6.2. DNN Models in Different Sub-Bands of Three 2 GHz Blocks

Although the unit direction vectors were used as the target outputs in the network training, az and el angles were still used to present the training, validation, and testing results.

All DNN training was conducted using PyTorch (2.13.0). Each training session employed an epoch size of 512. The Adam optimizer was used with an initial learning rate of 0.002. To manage learning rate adjustments, the ‘ReduceLROnPlateau’ scheduler was applied, configured with a reduction factor of 0.25, a patience of 25 epochs, and a threshold of 10^−5^. Training was automatically terminated if the learning rate dropped below 10^−6^ as dictated by the scheduler, or root mean square errors (RMSEs) in az and el angles were less than or equal to 0.5°.

To further demonstrate the flexibility of the DNN-based MOOD-DF method, different numbers of sub-bands were used within each 2 GHz block, as summarized in Table 7. In total, 14 MLP networks were trained for DF inference across the three 2 GHz frequency blocks, and all networks employed the same uniform architecture of 4×4096 MLPs without undergoing optimal MLP search, as we did in [58].

The choice of using two, four, and eight sub-bands in the low-, middle-, and high-frequency blocks, respectively, was motivated by the embedded-element far-field distributions presented in Appendix B. These distributions show that the directional patterns become increasingly irregular at higher frequencies, which makes DNN training more challenging. Reducing the sub-band bandwidth mitigates this effect and facilitates more stable and easier model training.

## 7. Performance Evaluation of the Proposed DF System

Utilizing simulated data samples and network frameworks from preceding sections, this section presents a comprehensive evaluation of the DNN-based DF sub-band models. It details the training characteristics, analyzes estimation accuracy across all 14 sub-bands, and quantifies forward inference latency on an NVIDIA RTX 6000 Ada GPU.

### 7.1. DNN Model Training Results in Different Sub-Bands

The network training performance for all 14 sub-bands is presented in Table 8, Table 9 and Table 10 for the low, middle, and high 2 GHz frequency blocks, respectively, where all data were generated at a targeted 10 dB SNR. Within each sub-band, separate models were trained using discrete dataset volumes of 5.0, 2.5, and 1.0 million samples to evaluate scaling behavior. To construct these training configurations, the samples were randomly selected from the total available data pool of that specific sub-band (in Table 7).

During network training, a corresponding 10% sample volume (i.e., 500k, 250k, or 100k samples) was independently drawn from the remaining data samples to serve as the validation data for early stopping and hyperparameter monitoring. Crucially, there was zero redundancy or sample overlap between the training and the validation sets. Finally, all remaining unused samples within the sub-band data samples were utilized as the independent test set.

From Table 8, Table 9 and Table 10, the following can be observed:

1. In general, the models provide more accurate estimations in az than in el.
2. As the number of training samples decreases, the overall RMSE increases; however, the models still achieve good az performance, with az RMSEs remaining below 1°.
3. When the number of training samples is reduced, the estimation error in el increases across all sub-bands.

### 7.2. DNN Model Testing Results of Different Sub-Bands

The sub-band models’ inference performance metrics are summarized in Table 11, Table 12 and Table 13 for the low, middle and high 2 GHz frequency blocks, respectively, evaluated at a targeted 10 dB SNR. Consistent with the training and validation characteristics, the az estimations of sub-band models exhibit better accuracy compared with those of el estimations across all sub-bands. On average, the models achieve a robust statistical confidence greater than 90% that the absolute az inference error remains less than 1.0°. This estimation profile degrades noticeably in el, where the probability of achieving below 1° decreases as the operating frequency increases. Furthermore, these tables document the forward inference latency, which remains at a consistently low microsecond level for all evaluated 4 × 4096 MLP sub-band models under batch execution.

A deeper comparative analysis of the data in these tables reveals the following critical insights:

- As the randomly selected training volume draws from 5.0 million down to 1.0 million samples within each sub-band, both az and el RMSEs predictably degrade. However, the az estimation demonstrates remarkable data resilience; even when constrained to 1.0 million samples, the az RMSE strictly preserves sub-degree accuracy across nearly all 14 evaluated sub-bands.
- The performance gap between az and el widens dramatically as a function of carrier frequency. In the 2–4 GHz frequency block (Table 11), allocating 5.0 million training samples allows the models to successfully bound both az and el RMSEs well below 0.5°. In the 16–18 GHz frequency block (Table 13), despite deploying the 5.0 million samples, the baseline el RMSE exhibits a persistent floor between approximately 0.472° and 1.037°, which sharply deteriorates up to 3.202° when only 1.0 million training samples were used. This underscores the intense geometric and scattering non-linearities present at shorter wavelengths.
- The percentage metrics (|az_error| ≤ 1° versus |el_error| ≤ 1°) demonstrate that el errors are not uniformly distributed. For example, in the 17.75–18.00 GHz sub-band with only 1.0 million training samples, the success rate for el drops to approximately 48.6%, while the corresponding az success rate remains above 91%. This large gap indicates that a substantial fraction of the el errors are significantly larger than 1°, even though the overall RMSE is only a few degrees. As discussed in Section 9.1, these larger errors are associated with el angles that are geometrically unfavorable due to the irregular vertical layout of the array.

To ensure complete statistical reporting and verify the repeatability of these deep learning models, 95% confidence intervals (CIs) are incorporated alongside every RMSE within Table 11, Table 12 and Table 13. Due to the large test dataset size in each sub-band, the statistical margin of error converges tightly around the mean, as can be observed from these tables.

### 7.3. Inference Results with Different Targeted SNRs

**Figure 13:**
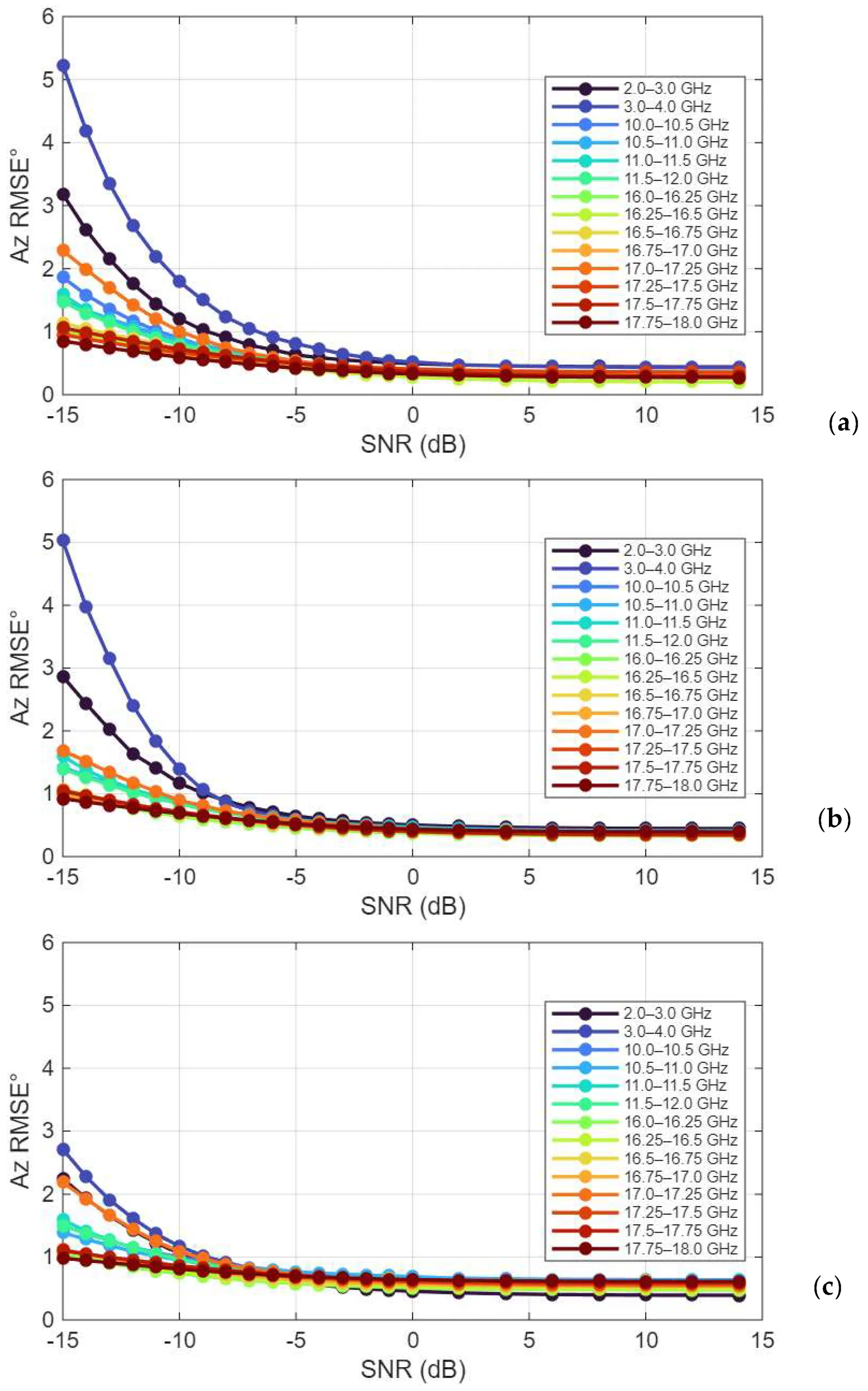
RMSE of az versus SNR for models trained with (**a**) 5.0, (**b**) 2.5, and (**c**) 1.0 million samples (top to bottom).

**Figure 14:**
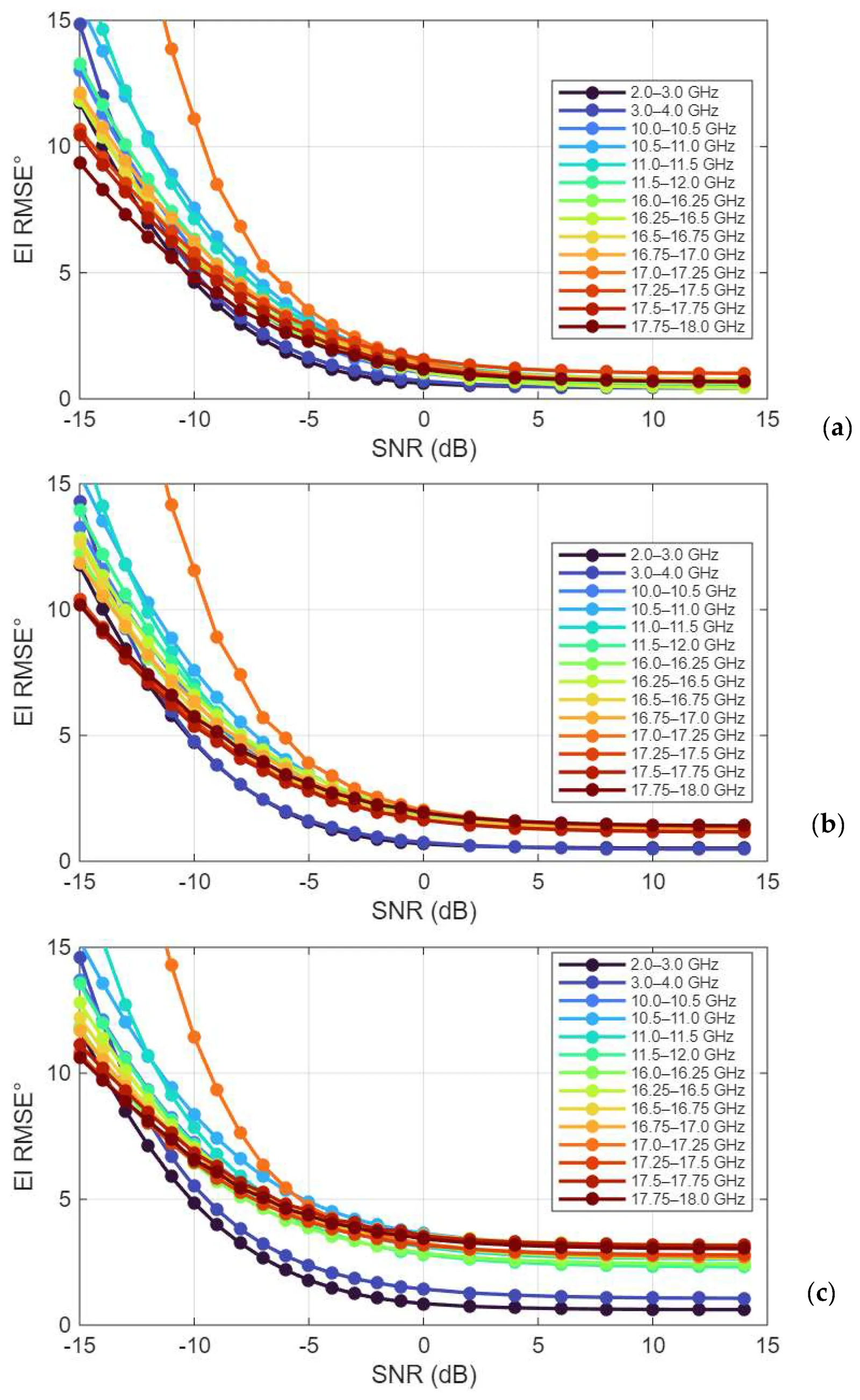
RMSE of el versus SNR for models trained with (**a**) 5.0, (**b**) 2.5, and (**c**) 1.0 million samples (top to bottom).

In the previous subsection, all testing results were obtained at a targeted SNR of 10 dB with the input of seven RF receivers. Using the sub-band models developed in the preceding section, the estimation performance under different targeted SNR levels is shown in Figure 13 and Figure 14 for az and el, respectively. From these figures, one can observe the following: (1) the models across all 14 sub-bands achieve less than 1.0° RMSE in az at an approximately −7.0 to −5.0 dB targeted SNR level, (2) as the number of training samples decreases, the el estimation accuracy also degrades, and (3) the estimation accuracy for az is consistently better than that for el.

## 8. DF System Performance Assessment Using CRLB and MUSIC Benchmarks

Because each sub-band model is trained using data from many frequencies and directions within its corresponding frequency sub-band, the proposed DF system inherently operates as a wideband DF estimator. Consequently, direct comparisons with conventional narrowband DF approaches, such as the CRLB and spectral MUSIC, involve an inherent paradigm mismatch. To provide intuitive and meaningful performance benchmarks, this section first compares the proposed DF system with the CRLB using both 3D isotropic and biconical manifolds at three representative frequencies selected from the three frequency blocks. Next, the degree of spatial-manifold aliasing is quantified at these representative frequencies to illustrate the ambiguity present in the 3D biconical manifold. Finally, the DF estimation performance of the proposed DF system is compared with that of the spectral MUSIC algorithm.

### 8.1. Performance Comparison with the CRLB at Selected Frequencies

Since the CRLB is typically evaluated for a narrowband signal at a specific frequency, an intuitive benchmark is established by comparing the DF performance of the proposed method with both isotropic-array manifold (isotropic-manifold) and biconical-manifold CRLBs at three representative frequencies 3.4938 GHz, 10.775 GHz, and 16.8988 GHz, corresponding to the low-, mid-, and high-frequency blocks, respectively.

To quantify the SNR actually seen by the DNN models in the frequency domain, referred to here as ${SNR}_{fd}$, a noise-free and biconical-manifold dataset (ASV_clean_) was generated on the grid az×el=[−180°:0.5°:179.5°]×[−50°:0.5°:50°] at an SNR of 100 dB, using the same model discussed in Section 4; then, noisy ASV_noisy_ data were generated on the same grid at each SNR∈[−15:0, 2:2:14] dB. In each direction on the grid, all ASVs were calculated using (9) at a given frequency. After pairing identical directions, the ${SNR}_{fd}$ is

(13) $${SNR}_{fd}=10\log_{10}\frac{{\left\|{ASV}_{clean}\right\|}^{2}}{{\left\|{ASV}_{noisy}-{ASV}_{clean}\right\|}^{2}}(\mathrm{median}\;\mathrm{over}\;\mathrm{the}\;\mathrm{grid}).$$

The realized processing gain, when the data are transferred from the time- to frequency-domain, is $G={SNR}_{fd}-SNR$, and the results are shown in Table 14.

The table shows that G is below the ideal 36 dB of $2^{12}$-point FFT processing gain. The discrepancy arises from several practical factors:

- Time-domain windowing loss, since a finite observation window is applied to the received signal.
- Spectral leakage, because the signal frequency is generally not located exactly at an FFT bin center.
- ASV normalization, where the frequency-domain ASV in (9) is normalized using the reference channel.

**Figure 15:**
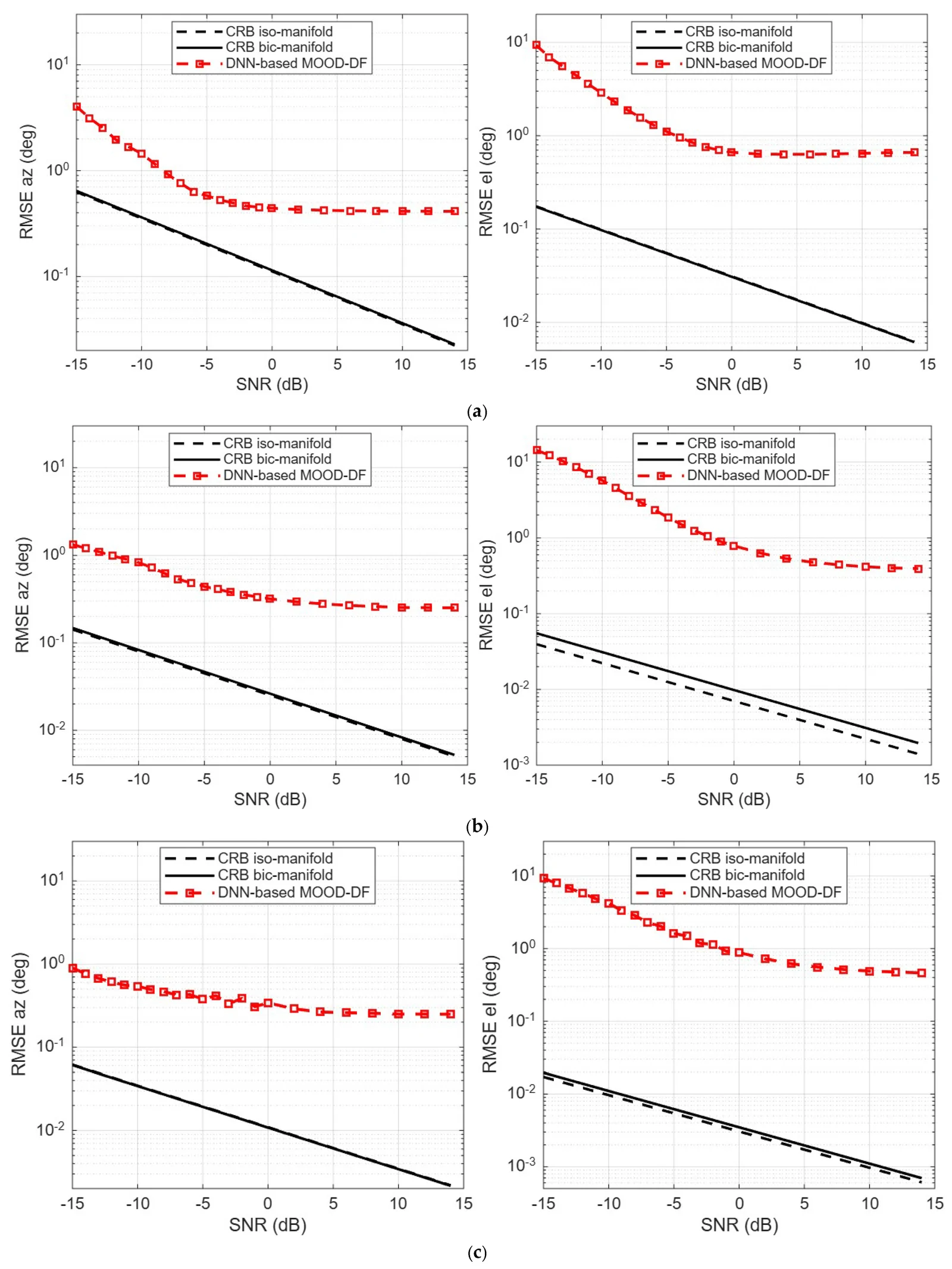
Estimation performance comparison between the proposed DF method and CRLBs: (**a**) 3.4938 GHz, (**b**) 10.775 GHz and (**c**) 16.8988 GHz.

Using $\sigma^{2}=10^{-(SNR+G)/10}$ in CRLB calculations, Figure 15 compares the DF estimation performance of the proposed method with the isotropic-manifold and biconical-manifold CRLBs.

Several observations can be made from Figure 15. First, the two CRLBs are very close to each other. After reference-channel normalization, the angle dependence of the biconical array ASV is mainly dominated by the same 3D geometric phase as the isotropic array; so, the Jacobian is set by the phase change with direction. Second, the CRLBs lie well below the proposed method RMSE at every frequency. This is because the physical aperture of the 3D array is much larger in terms of wavelength (see Table 15), especially at two high frequencies. The large array span in the z-direction results in a substantially lower CRLB in el than in az.

It is worth noting that the objective of network training was not to approach the CRLB. To prevent overfitting and maintain robust generalization performance over a large number of frequencies and directions, training was terminated once both validation az RMSE and el RMSE fell below 0.5°.

### 8.2. Evaluation of Spatial-Manifold Aliasing

A critical vulnerability of arbitrary, non-uniform 3D sparse array structures operating across an ultra-wideband spectrum is the susceptibility to dual-mode aliasing. First, frequency aliasing occurs across the multi-octave frequency footprint when distinct spectral segments generate overlapping phase behaviors. Within the proposed DF architecture, this issue is structurally eliminated by our sub-band partitioning scheme and the inclusion of the mapped frequency as a joint network input feature. Second, spatial aliasing occurs when separate arrival angles have the same ASV at a given frequency, rendering standard DF approaches ambiguous.

To investigate the manifold characteristics introduced by the physical geometry of the wideband array, an analytical examination of the biconical manifold was performed. Simulations were conducted using the noise-free steering vectors, ASV_clean_, generated on the direction grid at the three representative frequencies.

To quantify these spatial spots, a coherence threshold ρ=0.99 was applied to the ASVs of distinct directions. Adjacency on the direction graph was then gated by an elliptic spatial threshold whose axes are the ∆az and ∆el entries of Table 16. Those axes are the half-power main-lobe widths of equivalent uniformly illuminated line apertures of length $L_{xy}$ in az and $L_{z}$ in el, and they are used as in-beam versus out-of-beam thresholds. Two directions are treated as aliases when their ASV coherence ρ≥0.99 and their angular separation lies outside that ellipse (out-of-beam). Pairs that are highly coherent but remain inside the ellipse are counted as the “main lobe”.

It must be emphasized that these filter resolution thresholds are not unique; rather, they are selected as a representative baseline to illustratively demonstrate the inherent severity of spatial aliasing inside this non-uniform 3D array at the chosen frequencies. Table 17 compiles the spatial aliasing results on the on-grid biconical manifold. Of the 144,720 directions, 134,648, 132,593, and 141,133 have a unique ASV at 3.4938, 10.775, and 16.8988 GHz, respectively.

Based on the analysis above, it can be concluded that the 3D DF array exhibits severe spatial aliasing characteristics that can critically degrade the performance of conventional narrowband DF methods.

### 8.3. Performance Comparison with MUSIC at Selected Frequencies

To ensure a fair comparison, the spectral MUSIC algorithm was evaluated under a single-snapshot configuration identical to the ASV used by the DNN models for inference. Specifically, MUSIC was applied to a single realization of ASV_noisy_ in every direction of a total M=114,857 randomly picked directions, while the search process was performed over the corresponding biconical manifold. With this grid size, the results inherently have a 0.25° error.

**Figure 16:**
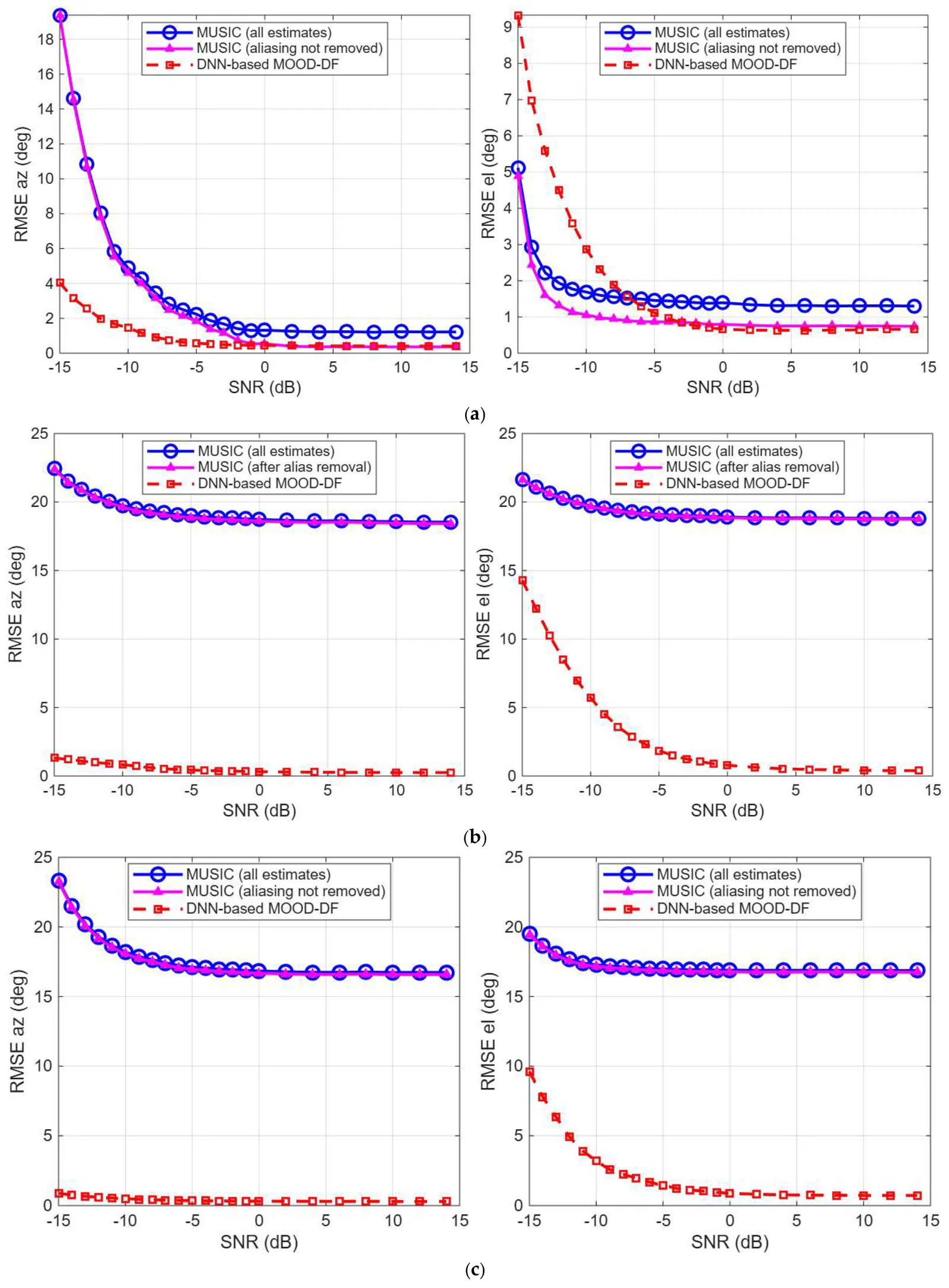
Estimation performance comparison between the proposed DF method and single-snapshot MUSIC: (**a**) 3.4938 GHz, (**b**) 10.775 GHz and (**c**) 16.8988 GHz.

The comparison results are shown in Figure 16. The MUSIC results indicate two distinct error mechanisms. At 3.4938 GHz, a noticeable reduction in both az and el RMSE is obtained after removing directions identified as spatial-manifold aliases, indicating that aliasing is a major source of estimation error at this frequency. In contrast, at 10.775 and 16.8988 GHz, alias removal provides only marginal improvement. This result suggests that the large MUSIC errors at the higher frequencies cannot be attributed solely to a number of identifiable alias directions. As the frequency increases, the embedded-element patterns become increasingly complex, exhibiting additional lobes, nulls, and more rapid angular variations (see Appendix B). Consequently, the corresponding array manifold becomes increasingly complex, giving rise to multiple competing pseudospectrum peaks. Under these conditions, MUSIC frequently converges to incorrect peaks even after the identified alias directions are excluded, resulting in persistent RMSE floors of approximately 19° and 17° at 10.775 GHz and 16.8988 GHz, respectively, in both azimuth and elevation. Therefore, the degradation in MUSIC performance at the higher frequencies appears to be dominated by the increased complexity of the biconical manifold.

It is worth noting that, as shown in Table 18, the sub-band models were trained using substantially fewer samples at the tested three frequencies than the 144,720 directions evaluated by MUSIC. Nevertheless, the proposed DF system achieves significantly lower RMSE than single-snapshot MUSIC at all three representative frequencies, demonstrating that the DNN-enabled estimator can effectively operate on the highly aliased and complex manifold of the modeled multi-octave biconical array.

## 9. Discussion

Based on the model training and testing results in the previous section, this section presents two discussions. First, the array architecture is analyzed from a 3D geometric perspective to explain why the az RMSE consistently outperforms that of el. Second, the applicability of the full-wave EM model to future hardware development and anechoic chamber measurements is discussed.

### 9.1. Geometric Factors Affecting Spatial Precision and Considerations for el Accuracy

The physical architecture of the seven-element 3D DF array was originally engineered with three primary design considerations:

- The utilization of non-uniform element positions to suppress spatial aliasing across a multi-octave spectrum.
- The minimization of mutual coupling by maximizing inter-element spatial separation relative to the physical volume of the wideband biconical elements.
- The mitigation of structural blockage from adjacent elements for incoming wave fronts arriving at steep vertical angles away from the XY plane.

These overlapping constraints resulted in the specific structural configuration analyzed in Section 3. Based on the coordinates detailed in Table 4, the physical bounds of the array occupy a bounding envelope spanning 223.7 mm × 204.08 mm in the horizontal plane, while extending across a significantly larger span of 790.50 mm along the vertical *Z*-axis.

When looking from a top view and projecting onto the XY plane, Element 1 serves as the central reference at the coordinate origin, while Elements 2 through 7 form a close approximation of a six-element uniform circular array (UCA). Conversely, when the array is viewed from the side, the peripheral elements present a highly structured, alternating vertical zigzag profile.

This structural distribution explains the performance asymmetry observed between the two orthogonal angular domains, i.e., az and el. In the horizontal plane, the quasi-UCA layout with a central reference anchor ensures that an incoming signal from any angle encounters a highly balanced and consistent baseline projection.

In contrast, when evaluating el angles, the effective spatial baseline varies dramatically, because different arrival angles experience completely different projections of the vertical zigzag profile. Although these unique projections provide distinct features for the DNN models, the elements are distributed over a much wider and more irregular spatial grid along the *Z*-axis compared to their tightly grouped, regular spacing in the XY plane. Consequently, the array structure possesses an inherent geometric anisotropy that naturally favors az estimation over el estimation.

To mitigate this structural imbalance and enhance el estimation accuracy in subsequent design iterations, the following three engineering strategies can be considered:

- Developing a next-generation wideband antenna element that maintains the omnidirectional “donut-shaped” 3D radiation pattern of the biconical antenna but shrinks its physical volume (e.g., reducing the height). Scaling down the physical element size directly reduces mutual scattering and adjacent structural blockage, allowing the total vertical *Z*-axis span to be compacted.
- Integrating additional antenna elements into the vertical zigzag stack to increase spatial sampling density along the *Z*-axis. This denser spatial grid expands the available phase-diversity features across complex el angles, providing the neural network with more distinct tracking indicators.
- As demonstrated by the data scaling benchmarks in Section 7.1, expanding the training dataset to 5.0 million samples per sub-band provides an immediate path to mitigating geometric non-linearities, successfully driving the el RMSE below 1.0° across nearly all evaluated sub-bands.

### 9.2. Implications for Future Hardware Prototyping and Experimental Validation

As established in Section 1, the end-to-end DF system model developed in this work provides a computational framework that supports future hardware prototyping and chamber validation. The integration of full-wave EM modeling, high-fidelity receiver simulation, and data-driven DF processing enables high-fidelity numerical performance assessment prior to hardware implementation and experimental testing. Furthermore, it enables array structure optimization for subsequent hardware prototyping and establishes a clear path forward for anechoic chamber data collection. Immediate methodological priorities prior to physical prototyping and chamber testing include the following: (1) Finding an optimal vertical element spacing, which requires balancing two opposing trade-offs: minimizing inter-element mutual coupling (which favors larger separation) and maximizing elevation estimation accuracy (which favors tighter spacing). (2) Chamber data collection, which requires optimizing the trade-off between angular sampling density and frequency resolution. This must account for the slow mechanical re-pointing speeds of the positioner versus rapid electronic frequency scanning. (3) Given that the rod-loaded biconical elements exhibit a dominant el polarization, methods to enhance the embedded-element az far-field component can be explored, such as integrating polarizers or controlled multi-reflectors.

## 10. Conclusions

This work presented a system-level evaluation of a multi-octave omnidirectional DF system using full-wave EM modeling of a seven-element biconical array and high-fidelity digital receiver simulation. By replacing the isotropic antenna elements and simplified receiver models used in the earlier study with physics-based antenna array and receiver models that incorporate mutual coupling, embedded-element radiation effects, receiver noise, cable length mismatches, and antenna–receiver impedance mismatches, the DF system was evaluated under conditions that provide a higher-fidelity representation of the antenna array and receiver environment. The results demonstrated that the proposed DF system remains effective across the investigated frequency blocks, achieving sub-degree azimuth RMSE and elevation RMSE below approximately 1.8° at SNRs near 0 dB while maintaining microsecond-scale inference latency suitable for near-real-time DF applications.

Benchmark comparisons with the CRLB and single-snapshot MUSIC provided theoretical and conventional performance references for the proposed DF system. Although a performance gap relative to the CRLB remains, the results showed that the system can successfully operate on the complex and highly spatially aliased three-dimensional manifold of the multi-octave biconical array. At 3.4938 GHz, a significant portion of the MUSIC error was attributed to spatial-manifold aliasing. In contrast, at 10.775 GHz and 16.8988 GHz, alias removal provided only limited improvement, indicating that MUSIC performance became increasingly constrained by the complexity of the embedded-element manifold and the resulting competing pseudospectrum peaks. Under these conditions, MUSIC exhibited RMSE floors of approximately 19° and 17°, respectively, whereas the proposed DF system maintained substantially lower estimation errors.

Unlike conventional DF approaches that rely on calibration measurements and correction tables to compensate for channel-to-channel variations, the proposed system incorporates these effects directly into the training data through the high-fidelity antenna array and receiver models. Consequently, receiver-channel nonuniformities become part of the learned frequency-dependent array response rather than impairments that must be explicitly removed prior to direction estimation. Overall, the developed end-to-end modeling and evaluation framework represents an important step in advancing omnidirectional multi-octave DF systems from idealized theoretical studies toward future hardware prototyping and experimental validation. It also provides a high-fidelity numerical methodology for evaluating DNN-enabled DF systems operating on complex wideband antenna array manifolds under high-fidelity EM and receiver models.

## Figures and Tables

**Table 1:** Progression from idealized DF system studies to future hardware prototyping and validation.

Feature	Previous Work [58]	This Work (High-Fidelity Modeling)	Future Work (Experimental)
Objective	Algorithm proof-of-concept	Bridging theory to hardware realism	Field validation and prototyping
Antenna Element	Ideal isotropic radiators	Realistic 2–18 GHz biconical antennas	Fabricated biconical array
EM Environment	Zero mutual coupling	Full-wave EM (coupling and feed structure effects)	Anechoic chamber and full-array field testing
Data Source	Ideal ASVs	Full-wave EM + high-fidelity receiver model	Measured hardware data
Receiver Characteristics	Simple model	7-channel wideband digital receiver model	Integrated RF-to-digital hardware
Significance	Established DNN-based DF framework for RESM	Physics-aware performance evaluation	Hardware validation and field evaluation

**Table 2:** The dimensions of the three-segment biconical antenna.

Dimension	Final Dimension (mm)
Cone 1 height	11.0
Cone 1 bottom radius	1.68
Cone 1 top radius	16.0
Cone 2 height	10.0
Cone 2 top radius	20.0
Cylinder height	10.0

**Table 3:** The minimum ratios between $E_{el}/E_{az}$ and $E_{el}/E_{r}$.

Freq (GHz)	Min $E_{el}/E_{az}$ (dB)	Min $E_{el}/E_{r}$ (dB)	Freq (GHz)	Min $E_{el}/E_{az}$ (dB)	Min $E_{el}/E_{r}$ (dB)
2	83.2	62.5	11	45.6	46.8
3	75.5	67.4	12	46.4	46.4
4	69.3	65.4	13	47.3	43.6
5	65.0	65.9	14	45.6	40.8
6	57.7	65.7	15	47.3	39.9
7	51.3	61.2	16	45.3	39.6
8	47.5	57.5	17	34.9	38.0
9	48.5	54.4	18	42.5	35.5
10	47.8	50.0			

**Table 4:** Locations of 7 elements.

Elements	X (mm)	Y (mm)	Z (mm)
1	0.00	0.00	0.00
2	55.14	95.51	131.60
3	57.50	−99.60	264.20
4	−110.34	0.00	396.50
5	−58.46	−101.26	−132.50
6	−59.37	102.82	−265.00
7	113.37	0.00	−394.00

**Table 5:** A list of frequency–element pairs for the 3D embedded-element field distributions shown in Appendix B, and the minimum values of the $\left|E_{el}/E_{az}\right|$ and ${|E}_{el}/E_{r}|$ ratios (dB) for each pair.

**Frequency (GHz)**	**Element**	Min ${|E}_{el}|/|E_{az}|$ (dB)	Min $\left|E_{el}|/|E_{r}\right|$ (dB)
2	1	14.2	41.9
3	2	15.9	48.8
4	3	15.8	47.8
5	4	17.1	45.5
6	5	9.9	50.9
7	6	9.8	46.3
8	7	9.7	45.5
9	7	13.9	45.6
10	6	13.2	45.9
11	5	11.0	45.1
12	4	17.4	42.7
13	3	15.5	42.8
14	2	15.8	42.4
15	1	16.3	41.1
16	3	16.8	38.3
17	4	19.1	37.6
18	7	18.0	32.5

**Table 6:** Parameters and data used in receiver (Rx) model.

**Parameters**	**Description**	Numbers
N	Channel number	7
B (GHz)	Receiver bandwidth	1.1
$f_{LO}$ (GHz)	LO frequency	$\frac{f_{1}\left(S\right)+f_{2}\left(S\right)}{2}-1.1$
T (K)	Absolute temperature	300
$k_{B}$ (J/K)	Boltzmann constant	1.38 × 10^−23^
NF (dB)	Receiver channel noise figure	[5.3, 4.9, 4.8, 4.8, 5.0, 5.1, 5.3]
l(n) (mm)	Cable length	[199.6, 199.8, 200.0, 200.1, 199.9, 200.3, 199.7]
$\Gamma_{A}(f,n)$	Antenna input return loss	Calculated in Section 3
$\Gamma_{R}(f,n)$	Receiver input port return loss	FR_MAAL-011130_5V_74mA.s2p [69]
SNR (dB)	Target SNR	10
$f_{s}$ (Samples/s)	ADC sampling rate	5.12 × 10^9^
*P*	FFT size	$2^{12}$

**Table 7:** Number of sub-bands in three 2 GHz blocks.

Band Limits	Number of Sub-Bands	Sub-Band Size (GHz)	Approximate Number of Samples in Each Sub-Band
2–4 GHz	2	1	72,130,196
10–12 GHz	4	0.5	36,065,098
16–18 GHz	8	0.25	18,032,549

**Table 8:** Training and validation performance of two sub-bands in 2–4 GHz.

Sub-Band	Training Data Points (mil)	Validation Data Points (mil)	Validation az_RMSE (°)	Validation el_RMSE (°)
2.00–3.00	5.0	0.50	0.43	0.43
2.00–3.00	2.5	0.25	0.44	0.50
2.00–3.00	1.0	0.10	0.39	0.54
3.00–4.00	5.0	0.50	0.42	0.42
3.00–4.00	2.5	0.25	0.38	0.44
3.00–4.00	1.0	0.10	0.48	1.03

**Table 9:** Training and validation performance of four sub-bands in 10–12 GHz.

Sub-Band	Training Data Points (mil)	Validation Data Points (mil)	Validation az_RMSE (°)	Validation el_RMSE (°)
10.00–10.50	5.0	0.50	0.26	0.58
10.00–10.50	2.5	0.25	0.39	1.26
10.00–10.50	1.0	0.10	0.63	2.63
10.50–11.00	5.0	0.50	0.27	0.66
10.50–11.00	2.5	0.25	0.39	1.27
10.50–11.00	1.0	0.10	0.62	3.12
11.00–11.50	5.0	0.50	0.30	0.84
11.00–11.50	2.5	0.25	0.39	1.29
11.00–11.50	1.0	0.10	0.56	2.70
11.50–12.00	5.0	0.50	0.28	0.70
11.50–12.00	2.5	0.25	0.37	1.29
11.50–12.00	1.0	0.10	0.54	2.31

**Table 10:** Training and validation performance of eight sub-bands in 16–18 GHz.

Sub-Band	Training Samples (mil)	Validation Samples (mil)	Validation az_RMSE (°)	Validation el_RMSE (°)
16.00–16.25	5.0	0.50	0.23	0.69
16.00–16.25	2.5	0.25	0.32	1.33
16.00–16.25	1.0	0.10	0.58	2.64
16.25–16.50	5.0	0.50	0.18	0.51
16.25–16.50	2.5	0.25	0.37	1.42
16.25–16.50	1.0	0.10	0.54	2.50
16.50–16.75	5.0	0.50	0.28	0.88
16.50–16.75	2.5	0.25	0.33	1.33
16.50–16.75	1.0	0.10	0.50	2.98
16.75–17.00	5.0	0.50	0.26	0.83
16.75–17.00	2.5	0.25	0.36	1.53
16.75–17.00	1.0	0.10	0.51	2.86
17.00–17.25	5.0	0.50	0.28	0.79
17.00–17.25	2.5	0.25	0.37	1.45
17.00–17.25	1.0	0.10	0.55	2.73
17.25–17.50	5.0	0.50	0.35	1.18
17.25–17.50	2.5	0.25	0.33	1.21
17.25–17.50	1.0	0.10	0.57	2.78
17.50–17.75	5.0	0.50	0.28	0.80
17.50–17.75	2.5	0.25	0.34	1.22
17.50–17.75	1.0	0.10	0.62	3.03
17.75–18.00	5.0	0.50	0.26	0.86
17.75–18.00	2.5	0.25	0.38	1.57
17.75–18.00	1.0	0.10	0.61	3.18

**Table 11:** Testing results of two sub-bands in 2–4 GHz. Three results in each sub-band corresponding to three different numbers of training samples in Table 8.

Sub-Band	az RMSE (°) & 95% CI	el RMSE (°) & 95% CI	% of |az_error| ≤1°	% of |el_error| ≤1°	Avg Latency (µs)
2.00–3.00	0.442 (0.441,0.443)	0.464 (0.446,0.484)	96.89	98.93	0.99
2.00–3.00	0.449 (0.447,0.450)	0.524 (0.503,0.548)	96.66	98.88	1.20
2.00–3.00	0.395 (0.393,0.399)	0.622 (0.599,0.645)	98.04	98.85	1.06
3.00–4.00	0.432 (0.431,0.432)	0.435 (0.422,0.447)	97.29	98.68	1.17
3.00–4.00	0.393 (0.392,0.394)	0.486 (0.472,0.502)	98.09	98.97	1.28
3.00–4.00	0.487 (0.485,0.489)	1.081 (1.058,1.103)	95.05	95.93	0.77

**Table 12:** Testing results of four sub-bands in 10–12 GHz. Three results in each sub-band corresponding to three different numbers of training samples in Table 9.

**Sub-Band**	az RMSE (°) & 95% CI	el RMSE (°) & 95% CI	% of |az_error| ≤1°	% of |el_error| ≤1°	Avg Latency (µs)
10.00–10.50	0.278 (0.277,0.278)	0.537 (0.528,0.546)	99.53	96.01	0.75
10.00–10.50	0.400 (0.399,0.402)	1.238 (1.221,1.254)	97.63	83.50	0.83
10.00–10.50	0.640 (0.637,0.644)	2.739 (2.719,2.757)	89.80	58.29	0.71
10.50–11.00	0.292 (0.291,0.292)	0.676 (0.660,0.694)	99.47	94.51	0.77
10.50–11.00	0.414 (0.413,0.415)	1.279 (1.266,1.292)	97.55	80.00	0.73
10.50–11.00	0.638 (0.636,0.639)	3.158 (3.139,3.177)	89.70	48.85	0.81
11.00–11.50	0.327 (0.326,0.328)	0.796 (0.788,0.805)	99.14	90.22	0.78
11.00–11.50	0.397 (0.396,0.393)	1.248 (1.234,1.261)	97.72	82.00	0.75
11.00–11.50	0.561 (0.559,0.562)	2.657 (2.640,2.674)	92.91	59.00	0.76
11.50–12.00	0.302 (0.301,0.302)	0.669 (0.663,0.675)	99.39	93.04	0.82
11.50–12.00	0.386 (0.386,0.387)	1.306 (1.293,1.320)	98.04	79.30	0.81
11.50–12.00	0.546 (0.545,0.548)	2.341 (2.326,2.356)	93.36	61.02	0.65

**Table 13:** Testing results of eight sub-bands in 16–18 GHz. Three results in each sub-band corresponding to three different numbers of training samples in Table 10.

**Sub-Band**	az RMSE (°) & 95% CI	el RMSE (°) & 95% CI	% of |az_error| ≤1°	% of |el_error| ≤1°	Avg Latency (µs)
16.00–16.25	0.279 (0.278,0.280)	0.631 (0.619,0.645)	99.63	93.51	0.72
16.00–16.25	0.330 (0.329,0.331)	1.241 (1.222,1.263)	98.95	81.73	0.61
16.00–16.25	0.479 (0.478,0.481)	2.453 (2.431,2.478)	95.63	59.63	0.68
16.25–16.50	0.208 (0.207,0.209)	0.472 (0.460,0.485)	99.89	96.99	0.71
16.25–16.50	0.400 (0.399,0.401)	1.397 (1.381,1.413)	98.13	74.23	0.64
16.25–16.50	0.492 (0.490,0.493)	2.699 (2.677,2.724)	96.36	55.91	0.73
16.50–16.75	0.306 (0.305,0.307)	0.787 (0.772,0.802)	99.36	92.11	0.74
16.50–16.75	0.340 (0.338,0.341)	1.251 (1.230,1.273)	98.83	82.61	0.71
16.50–16.75	0.616 (0.613,0.619)	3.202 (3.176,3.232)	91.23	48.98	0.73
16.75–17.00	0.292 (0.290,0.293)	0.723 (0.701,0.749)	99.56	91.96	0.75
16.75–17.00	0.386 (0.384,0.388)	1.354 (1.337,1.370)	98.15	76.33	0.71
16.75–17.00	0.540 (0.535,0.544)	2.788 (2.759,2.816)	94.07	55.85	0.70
17.00–17.25	0.303 (0.302,0.304)	0.703 (0.688,0.719)	99.29	93.55	0.72
17.00–17.25	0.382 (0.381,0.384)	1.393 (1.365,1.423)	98.04	80.84	0.76
17.00–17.25	0.550 (0.547,0.553)	2.747 (2.710,2.785)	93.26	60.78	0.73
17.25–17.50	0.361 (0.360,0.362)	1.037 (1.014,1.061)	98.61	84.94	0.69
17.25–17.50	0.345 (0.344,0.346)	1.185 (1.157,1.216)	98.65	85.96	0.72
17.25–17.50	0.571 (0.569,0.573)	2.809 (2.779,2.840)	92.55	57.01	0.66
17.50–17.75	0.294 (0.293,0.295)	0.708 (0.692,0.728)	99.30	93.36	0.69
17.50–17.75	0.349 (0.348,0.351)	1.192 (1.168,1.216)	98.49	85.97	0.71
17.50–17.75	0.598 (0.595,0.600)	3.185 (3.150,3.221)	92.03	53.91	0.76
17.75–18.00	0.275 (0.274,0.276)	0.705 (0.693,0.717)	99.51	93.20	0.68
17.75–18.00	0.396 (0.394,0.398)	1.440 (1.418,1.462)	97.79	78.45	0.75
17.75–18.00	0.614 (0.612,0.616)	3.056 (3.031,3.081)	91.14	48.55	0.68

**Table 14:** Statistics of the measured processing gain at three representative frequencies.

Frequency (GHz)	Median Gain (dB)	Std Gain (dB)	Min Gain (dB)	Max Gain (dB)
3.49380	29.60	0.38	28.83	30.04
10.7750	29.98	0.19	29.76	30.41
16.8988	31.93	0.16	31.75	32.32

**Table 15:** The 3D DF array size versus wavelength, where $L_{xy}$ is the longest baseline in the XY plane, and $L_{z}$ is the array span in the z-direction.

Frequency (GHz)	$L_{xy}/\lambda$	$L_{z}/\lambda$
3.49380	~2.7	~9.2
10.7750	~8.4	~28.4
16.8988	~13.2	~44.6

**Table 16:** In-beam vs. out-of-beam angle filter thresholds.

Frequency (GHz)	∆az (Degree)	∆el (Degree)
3.4938	19.5	5.5
10.775	6.3	1.8
16.8988	4.0	1.1

**Table 17:** Spatial aliasing results on the on-grid biconical manifold (ASV_clean_) using coherence ρ=0.99 and the in-beam versus out-of-beam angle thresholds in Table 16; the total number of directions in the manifold is 144,720.

Frequency (GHz)	Total Groups	Single-Tons	Alias Groups (Size ≥ 2)
3.4938	135,395	134,648	747
10.7750	137,364	132,593	4771
16.8988	141,133	137,834	3299

**Table 18:** Sample counts at the three test frequencies for the sub-band models trained with 5, 2.5, and 1 million samples.

Frequency (GHz)	Sub-Band	Sample Count in Sub-Model Trained by 5 Million Samples	Sample Count in Sub-Model Trained by 2.5 Million Samples	Sample Count in Sub-Model Trained by 1 Million Samples
3.4938	3–4	7690	3911	1532
10.7750	10.5–11	16,287	8056	3237
16.8988	16.75–17	35,934	17,975	7122

**Figure A1:**
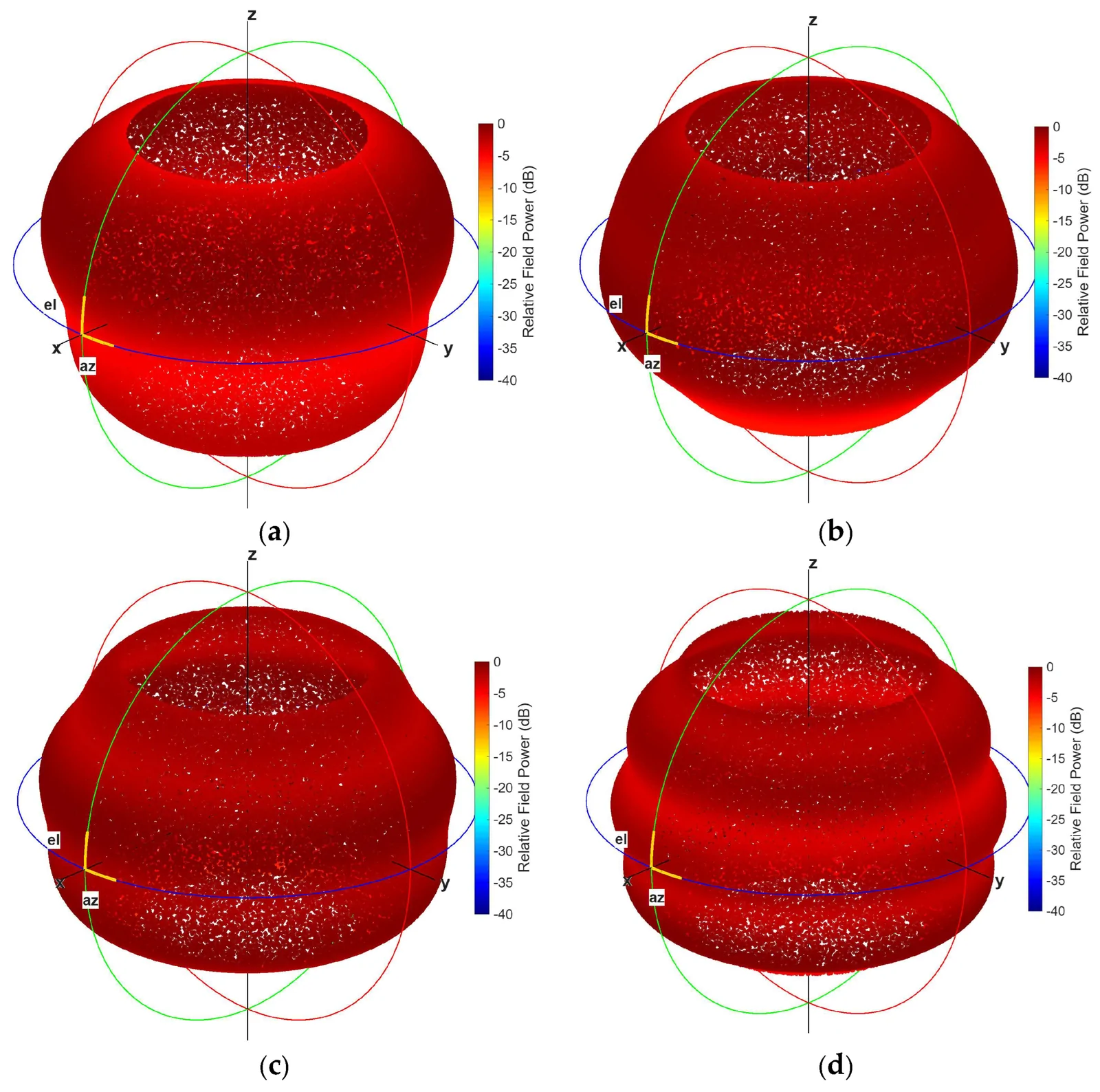

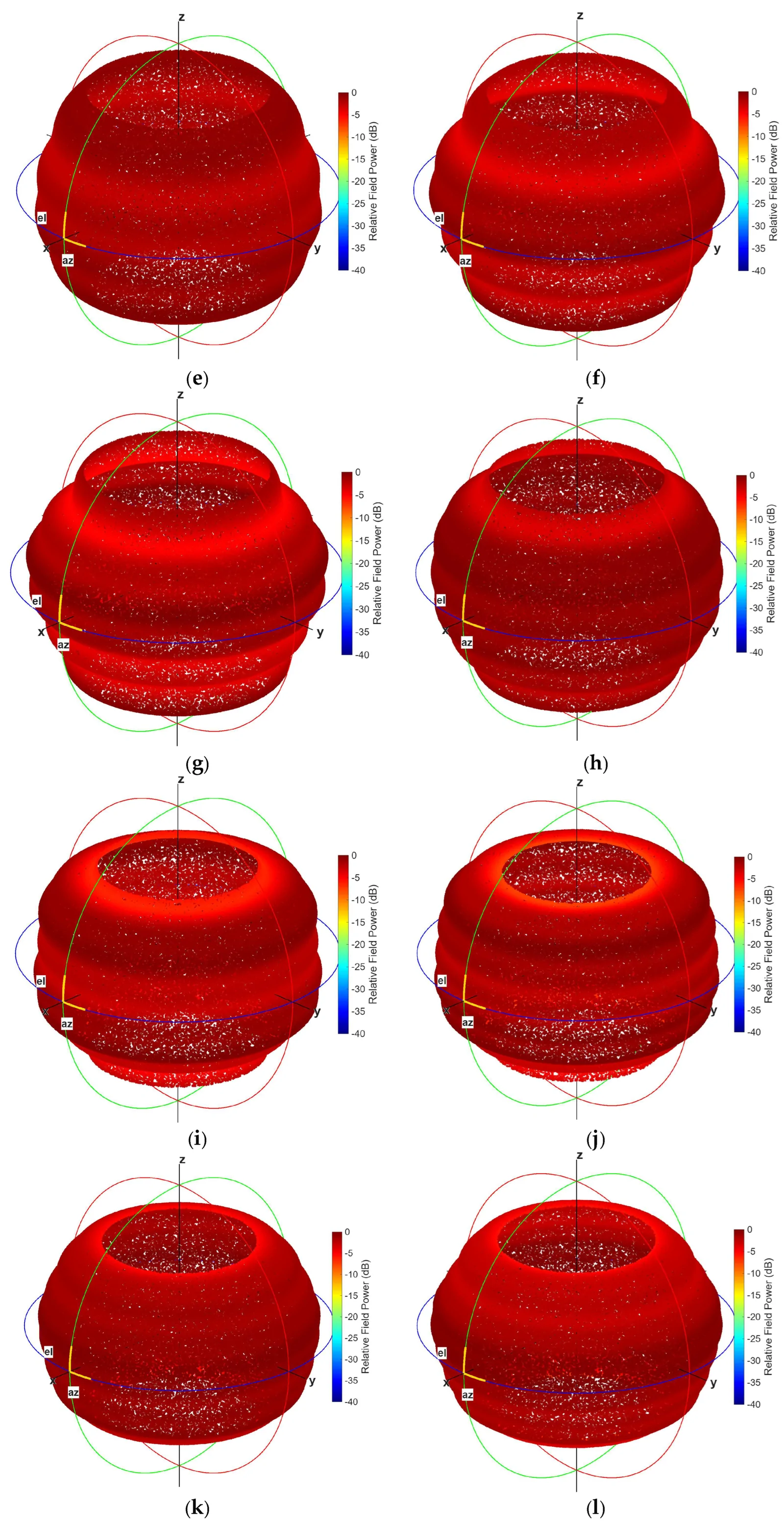

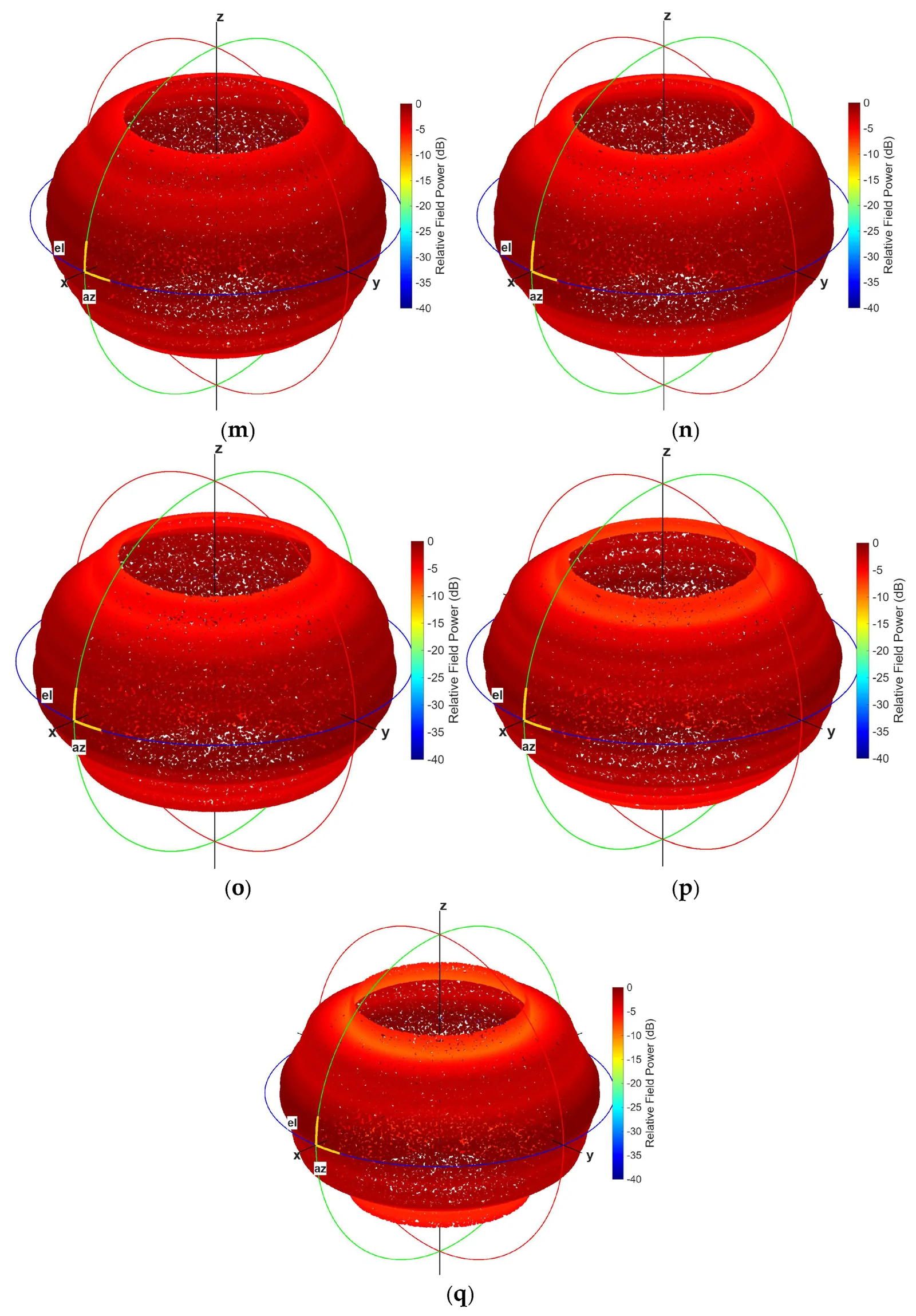
Normalized $E_{el}$ distributions of the biconical antenna. Data represent a 100 m far-field radius at randomly sampled directions within ±180° in az and ±50° in el for the 17 frequencies listed in Table 3; (**a**–**q**) are 2 to 18 GHz.

**Figure A2:**
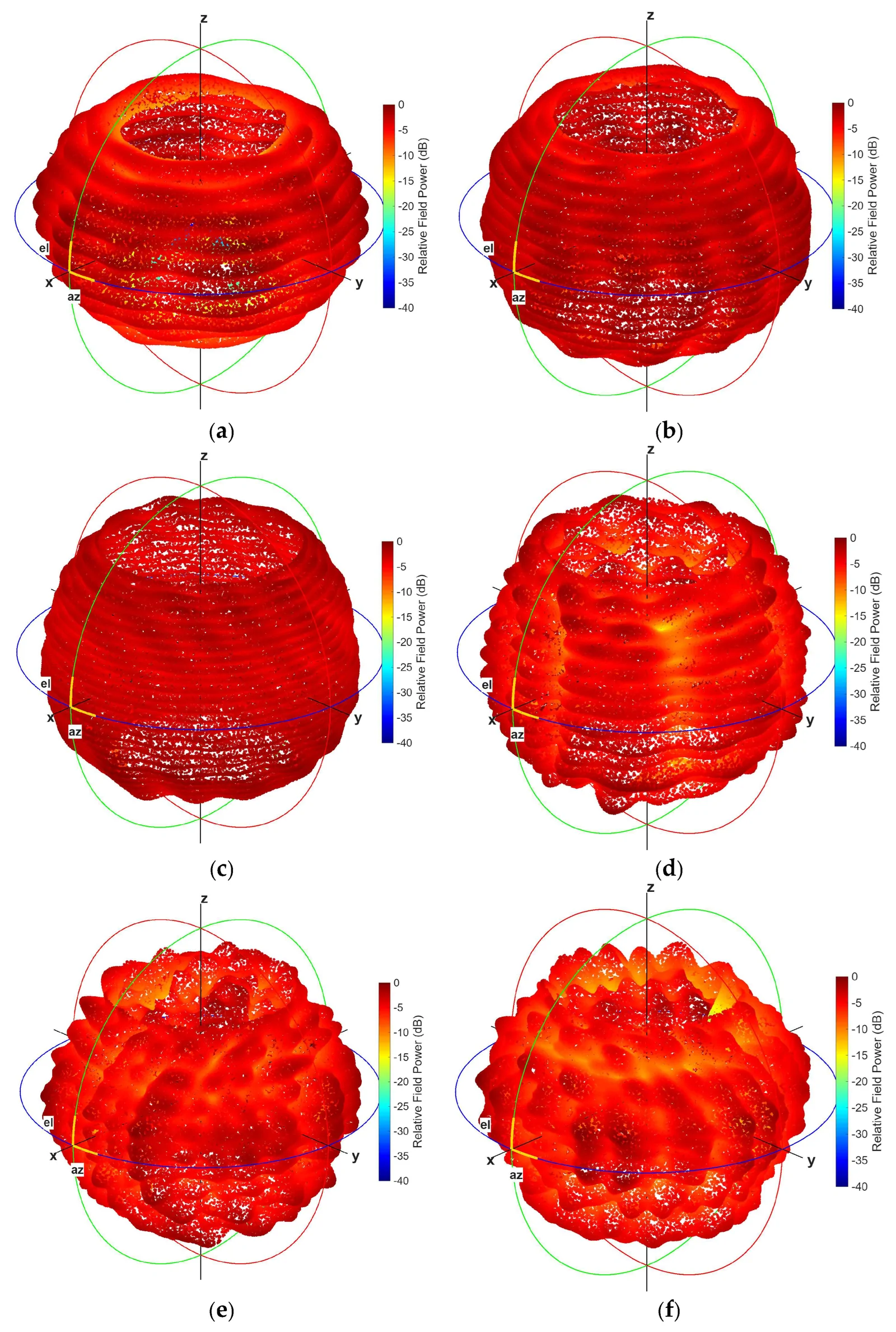

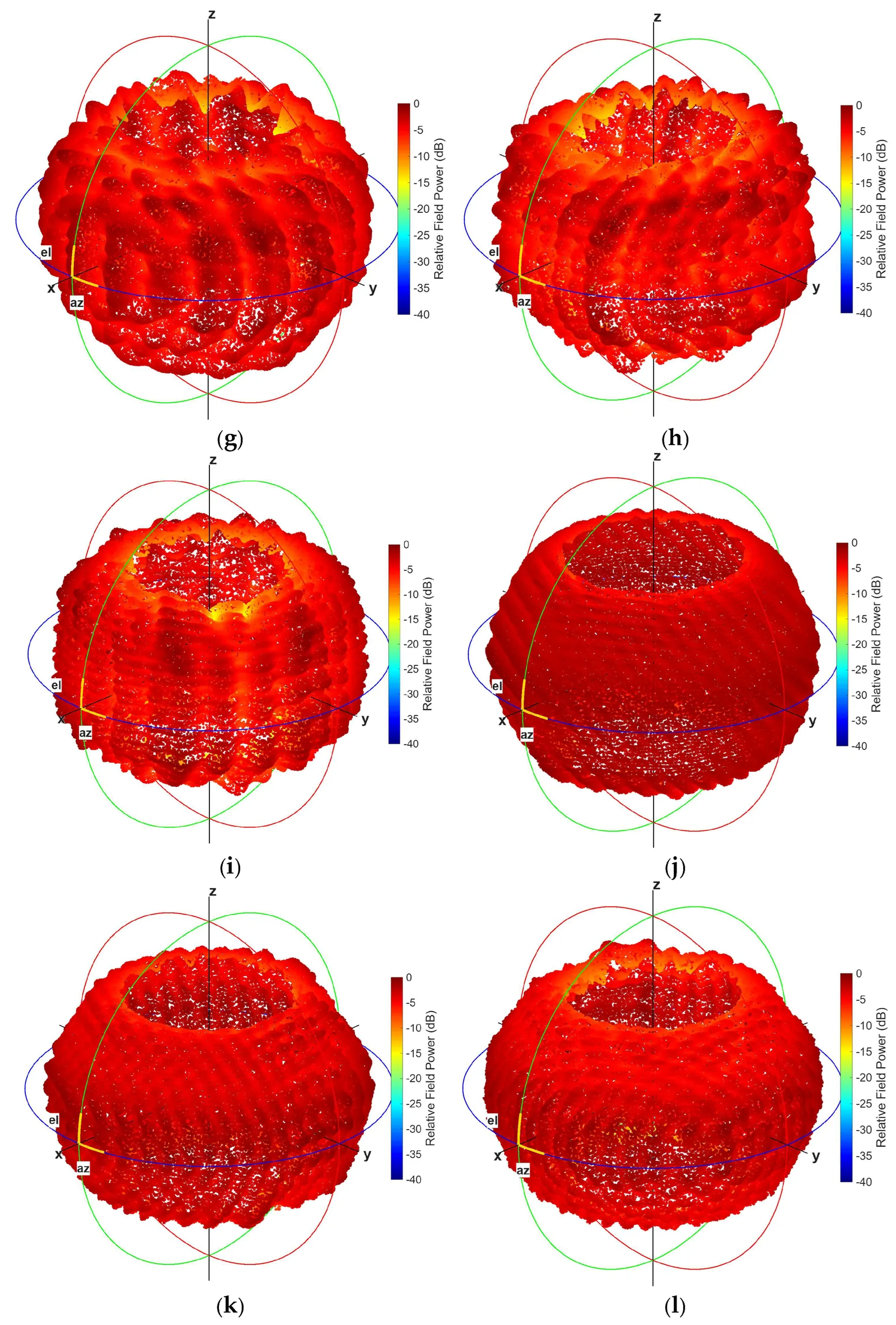

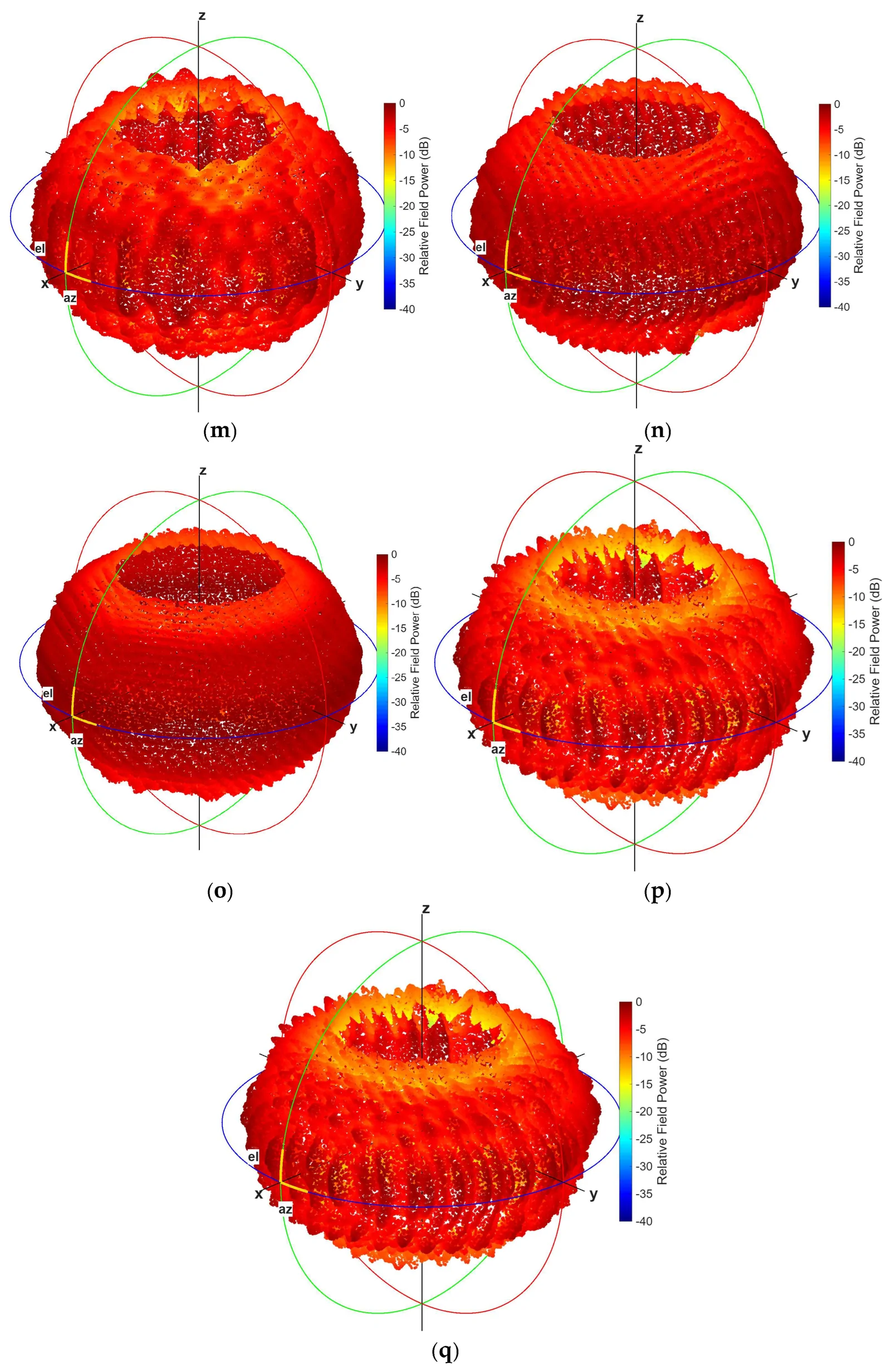
Normalized embedded-element $E_{el}$ distributions for the 7-element biconical array at randomly sampled directions within ±180° in az, ±50° in el and R=100 m. Subplots (**a**–**q**) show the 3D far-field distributions for the frequency–element pairs from (2 GHz, element 1) through (18 GHz, element 7), as listed in Table 5.

## Data Availability

The full-wave array steering vectors and trained network weights are not publicly available due to institutional and export control restrictions. The geometry of the array, receiver model parameters, and all metrics needed to interpret the results are contained in the article.

## Abbreviations

The following abbreviations are used in this manuscript:

1D	1-Dimensional
2D	2-Dimensional
3D	3-Dimensional
az	Azimuth
AOA	Angle of Arrival
ASV	Array Steering Vector
CI	Confidence Interval
CNN	Convolutional Neural Network
CS	Compressive Sensing
CRLB	Cramér–Rao Lower Bound
DDD	Direct Data-Domain
DF	Direction-Finding
DNN	Deep Neural Network
DOA	Direction of Arrival
el	Elevation
EM	Electromagnetic
ESM	Electronic Support Measures
ESPRIT	Estimation of Signal Parameters via Rotational Invariance Techniques
FFT	Fast Fourier Transform
FMCW	Frequency-Modulated Continuous Wave
GPU	Graphics Processing Unit
IF	Intermediate Frequency
LNA	Low Noise Amplifier
MIMO	Multiple-Input Multiple-Output
MLP	Multi-Layer Perceptron
MUSIC	Multiple Signal Classification
MoM	Method of Moments
MOOD-DF	Multi-Octave Omnidirectional Direction-Finding
NF	Noise Figure
ReLU	Rectified Linear Unit
REW	Radar Electronic Warfare
RESM	Radar ESM
RF	Radio Frequency
RMSE	Root Mean Square Error
SNR	Signal-to-Noise Ratio
TEM	Transverse Electromagnetic (wave)
UCA	Uniform Circular Array
VEL	Vector Effective Length

## Appendix A. Three-Dimensional Field Distributions in the Three-Segment Biconical Element Far Field

This appendix details the spherical-coordinate *E*-field distributions for the rod-loaded three-segment biconical element (see Figure 2) at a 100 m radius. Given the element’s linear polarization, the results are limited to the $E_{el}$ component, and only show el within [−50 50] deg.

It should be noted that, unlike conventional 3D radiation patterns that exhibit a continuous, smooth surface, the 3D plots contain minor discontinuities or “missing” areas. This effect is a result of the visualization method; the plots are generated using the *scatter3* function in MATLAB, which renders discrete data points. Because these plots are based on uniform, randomly sampled directions rather than a structured grid or interpolated mesh, the density of the points may vary slightly across the spherical surface.

## Appendix B. The Embedded Far-Field Distributions of Selected Elements at Specified Frequencies

The spherical-coordinate embedded far-field distributions of the elements in the seven-element biconical array (see Figure 5) at a radius of 100 m are provided in this appendix. The field data are limited to the $E_{el}$ component, which is the dominant ***E***-field component in the far-field region, and are shown only for el angles within [−50°, 50°]. The following can be found:

- Compared with the single-element far-field patterns presented in Appendix A, the embedded-element far-field patterns exhibit substantially greater ripple structure due to element interactions within the array.
- As frequency increases, the embedded-element patterns become increasingly complex, exhibiting a larger number of local maxima and nulls across the spatial domain.
